# Angiotensin II synergizes with BAFF to promote atheroprotective regulatory B cells

**DOI:** 10.1038/s41598-017-04438-6

**Published:** 2017-06-23

**Authors:** Padmapriya Ponnuswamy, Jeremie Joffre, Olivier Herbin, Bruno Esposito, Ludivine Laurans, Christoph J. Binder, Thomas F. Tedder, Lynda Zeboudj, Xavier Loyer, Andreas Giraud, Yujiao Zhang, Alain Tedgui, Ziad Mallat, Hafid Ait-Oufella

**Affiliations:** 1 Institut National de la Santé et de la Recherche Médicale (INSERM), Unit 970, Paris Cardiovascular Research Center, Université René Descartes Paris, Paris, France; 2 Department of Immunology, Duke University Medical Center, Durham, NC 27710 USA; 3 Center for Molecular Medicine (CeMM) of the Austrian Academy of Sciences and Department of Laboratory Medicine, Medical University of Vienna, 1090 Vienna, Austria; 4 Division of Cardiovascular Medicine, University of Cambridge, Addenbrooke’s Hospital, Cambridge, CB2 2QQ England UK; 5 Assistance Publique–Hôpitaux de Paris, Saint-Antoine Hospital, Université Pierre-et-Marie-Curie, Paris, France

## Abstract

Angiotensin II (AngII) promotes hypertension, atherogenesis, vascular aneurysm and impairs post-ischemic cardiac remodeling through concerted roles on vascular cells, monocytes and T lymphocytes. However, the role of AngII in B lymphocyte responses is largely unexplored. Here, we show that chronic B cell depletion (*Baffr* deficiency) significantly reduces atherosclerosis in *Apoe* ^−/−^ mice infused with AngII. While adoptive transfer of B cells in *Apoe* ^−/−^ */Baffr* ^−/−^ mice reversed atheroprotection in the absence of AngII, infusion of AngII in B cell replenished *Apoe* ^−/−^ */Baffr* ^−/−^ mice unexpectedly prevented the progression of atherosclerosis. Atheroprotection observed in these mice was associated with a significant increase in regulatory CD1d^hi^CD5^+^ B cells, which produced high levels of interleukin (IL)-10 (B10 cells). Replenishment of *Apoe* ^−/−^ */Baffr* ^−/−^ mice with *Il10* ^−/−^ B cells reversed AngII-induced B cell-dependent atheroprotection, thus highlighting a protective role of IL-10^+^ regulatory B cells in this setting. Transfer of AngII type 1A receptor deficient (*Agtr1a* ^−/−^) B cells into *Apoe* ^−/−^ */Baffr* ^−/−^ mice substantially reduced the production of IL-10 by B cells and prevented the AngII-dependent atheroprotective B cell phenotype. Consistent with the *in vivo* data, AngII synergized with BAFF to induce IL-10 production by B cells *in vitro* via AngII type 1A receptor. Our data demonstrate a previously unknown synergy between AngII and BAFF in inducing IL-10 production by B cells, resulting in atheroprotection.

## Introduction

Despite decades of research and availability of various cholesterol lowering drugs, atherosclerosis remains the major cause of mortality in the world. Beyond the undisputable role of low-density lipoproteins (LDL) in disease initiation^1^, a substantial body of evidence point to an important role of immune mediated mechanisms in disease progression^2, 3^. Central to both atherosclerosis initiation and progression is the renin-angiotensin system, which plays a crucial role through several distinct mechanisms^4^. Angiotensin II (AngII), generated from Angiotensin I by Angiotensin Converting Enzyme (ACE), is the primary effector molecule of the renin-angiotensin system and is known to cause vascular cell dysfunction/activation, predisposing the vascular wall to inflammatory cell recruitment^5–7^. AngII controls various physiological and pathological functions^8^, and its role has been extended to the innate and adaptive immune systems where it modulates macrophage polarization^9^, T lymphocyte activation^10^, and the balance of helper T cell subsets^11^. Other studies unveiled a pivotal immune-modulatory role of the renin-angiotensin system in autoimmune diseases and in patients with heart failure^12, 13^. In those studies, blockade of AngII signaling suppressed auto-reactive Th1 and Th17 responses, promoted regulatory T cells^12^, or led to reduction of Th1/Th2 ratio and inflammatory cytokine production^13^.

AngII-induced atherosclerosis is mediated through type 1A receptor (Agtr1a) signaling in vascular cells^14^. Invalidation of AngII signalling in bone marrow-derived leukocytes plays a minor role^14^ suggesting distinct roles of AngII on immune cell subsets. Indeed, while AngII induces T cell activation and proliferation, Agtr1a activation in macrophages has recently been shown to suppress their M1 pro-inflammatory phenotype, providing a protection in a mouse model of kidney injury^15^. The effects of AngII on B cell functions remain unknown. In the last decade, B cells were considered atheroprotective^16, 17^. More recently, we and others have reconsidered and redefined the role of B cells in atherosclerosis^18, 19^. The natural IgM secreting B1a subset was indeed shown to be atheroprotective^20, 21^. Conversely however, depletion of mature B2 cells using CD20 monoclonal antibody or genetic B2 cell deficiency in *Baffr* ^−/−^ mice resulted in reduced lesion development, indicating a proatherogenic role of the B2 cell subset^18, 19, 22^. The results are consistent with the pro-atherogenic role of T follicular helpher/germinal center B cell activation^23^. Still, the B2 cell subset is a heterogeneous population of folicular B cells, innate-like marginal zone B cells and regulatory B cells (Bregs) with distinct developmental and (patho)physiological functions, and their selective roles in modulating the immune response during atherosclerosis are yet to be defined. Two studies recently addressed the role of regulatory B cells in hyperlipidemia-associated vascular disease. While we showed that endogenous IL-10 producing B cells do not alter the development of atherosclerosis in hypercholesterolemic *Ldlr* ^−/−^ mice^24^, another study reported IL-10-dependent protection against neointima formation after exogenous supplementation with lymph node-derived regulatory B cells^25^. The discrepancy between the two studies can be explained by important differences in the disease models and the source, state of activation and function of the B cells used in the experiments. Thus, additional studies are required to better understand the mechanisms that promote the development and maintenance of atheroprotective regulatory B cells. We became interested in a recent study by Saussine *et al*. that reported high levels of IL-10 producing B cells in active sarcoidosis, a finding that was associated with elevated levels of BAFF and ACE activity^26^. Given the importance of ACE activity in patients with coronary artery disease^27^, we attempted to understand the role of AngII (in combination with BAFF) in modulating B cell-mediated immune responses. We used *Apoe* ^−/−^ mice infused with AngII, a well-established model of AngII-induced vascular disease, to understand the role of AngII in modulating B cell function during the development of atherosclerosis. To address the selective effect of AngII on B2 cells, we adoptively transferred mature B cells into B2 cell-deficient *Apoe* ^−/−^ */Baffr* ^−/−^ mice. The *Apoe* ^−/−^ */Baffr* ^−/−^ model also has the advantage of displaying high levels of BAFF, and is particularly suited to address the impact of the interaction between AngII and BAFF on the function of mature B cells. Here, we report the surprising finding that AngII signals through Atgr1a on B cells to induce a B10 regulatory and anti-atherogenic phenotype, particularly in a high BAFF environment.

## Materials and Methods

### Animals

Experiments were conducted according to the European Community for experimental animal use guidelines (L358-86/609EEC) and were approved by the Ethical Committee of INSERM. All mice used in the experiments were male on a C57BL/6J background and maintained on a chow diet. *Baffr* ^−/−^, *Il10* ^−/−^ and *Agtr1a* ^−/−^ mice were obtained from Jackson Laboratories and were backcrossed more than 10 generations into a C57BL/6J background. CD45.1 and *Apoe* ^−/−^ mice were from Charles River. *Apoe* ^−/−^ mice were crossed with *Baffr* ^−/−^ mice to generate *Apoe* ^−/−^ */Baffr* ^−/−^ mice. B cells were isolated from Wild-type (WT, C57BL/6J), *Il10* ^−/−^ or *Agtr1a* ^−/−^ mice using negative isolation kit from Miltenyi, according to manufacturer’s protocol. Recipient mice were injected with ~30 × 10^6^ B cells 3–6 weeks before AngII infusion. AngII was infused subcutaneously in 12–14 weeks old mice via osmotic pumps at the concentration of 1 μg/kg/min for 7 or 28 days.

### Atherosclerotic lesion

Atherosclerotic lesion assessment was performed on aortic sinus sections of mice infused with AngII for 28 days or in similar age matched controls without AngII infusion. Animals that died before 28 days of AngII (or PBS) infusion were not analyzed. Plasma cholesterol was measured using a commercial cholesterol kit (Sigma). Quantification of lesion size was performed as described earlier^18^. In brief, the basal half of the ventricles and the ascending aorta were perfusion-fixed *in situ* with 4% paraformaldehyde. After then, they were removed, transferred to a PBS-30% sucrose solution, embedded in frozen OCT and stored at −70 °C. Serial 10-μm sections of the aortic sinus with valves (80 per mouse,) were cut on a cryostat, as previously described^28^. Of every 5 sections, one was kept for plaque size quantification after Oil red O staining. Thus, 16 sections spanning 800 μm stretch of the aortic root were used to determine mean lesion area for each mouse. Oil Red O positive lipid contents were quantified by a blinded operator using HistoLab software (Microvisions). Plasma cholesterol was measured using a commercial cholesterol kit (Biomerieux).

### Systolic Blood Pressure Measurement

Systolic Blood Pressure (SBP) was measured in conscious mice using a tail cuff system (BP-2000 Visitech Systems), as previously described^29^. Measurements were always performed in the morning. In each animal, the system automatically performed 4 measurements first, which were not recorded, then, 10 consecutive measurements of SBP that were recorded. To avoid procedure-induced anxiety, and in each series of experiments, mice were accustomed to the tail cuff system during 3 consecutive days before basal SBP was recorded for 2 to 3 days (values were averaged) just prior mini-pump implantations. Then, SBP was measured at days 7, 14, 21 and 28, post-implantation.

### Cell culture

B cells were isolated from splenocytes by negative selection using a cocktail of antibody coated magnetic beads (Miltenyi Biotec, Germany), and the purity was confirmed to be >95%. Purified B cells were stimulated *in vitro* with anti-CD40/IgM or LPS for 72 h. The supernatant was stored for ELISA, and for intracellular staining of IL-10, the cells were stimulated with a leukocyte activation cocktail containing golgi stop for the last 5 hours of culture before flow cytometric analysis.

### Flow Cytometry

Single cell preparations of murine splenocytes were stained with the following fluorochrome conjugated antibodies: CD19-APC (clone: 1D3) B220-Amcyan (Clone: RA3-6B2), CD5-APC (Clone: 53–7.3), CD44-APC (Clone: IM7), CD45.1-PerCP-Cy5.5 (Clone: A20), CD4-FITC (Clone: RM4-5), CD3-PerCP-Cy5.5 (Clone: 145-2C11), CD23-PE (Clone: B3B4), CD21-PECy7 (Clone: 7G6), CD1d-Brillant Violet 450 (Clone: 1B1). For intracellular cytokine staining, lymphocytes were stimulated *in vitro* with leukocyte activation cocktail (BD) according to the manufacturer’s instructions for 4 h. Surface staining was performed before permeabilization using an intracellular staining kit (eBioscience). Intracellular IL-10 and IFN-γ was detected using IL-10-APC (Clone: JES5-16E3) and IFNγ-FITC (Clone: XMG1.2) antibodies, respectively.

### ELISA

B cells were isolated from *Apoe* ^−/−^ and *Apoe* ^−/−^ */Baffr* ^−/−^ mice transferred with B cells with or without AngII for 7 days or 28 days. Isolated B cells were stimulated *in vitro* with LPS or anti-CD40/IgM to determine IL-10 production. Cell free culture supernatants were frozen at −80 °C and then later examined for IL-10 production using a BD mouse IL-10 ELISA kit. sBAFF was determined by ELISA (R&D Systems) in the plasma samples from *Apoe* ^−/−^ and *Apoe* ^−/−^ */Baffr* ^−/−^ mice with or without B cell transfer. Circulating antibody titers against MDA-LDL and CuOx-LDL were determined by chemiluminescent ELISA, as previously described^30–32^.

### qPCR

RNA was isolated from B cells from *Apoe* ^−/−^ */Baffr* ^−/−^ mice transferred with B cells with or without AngII infusion. B cells isolated from WT or *Agtr1a* ^−/−^ mice were treated *in vitro* with AngII (10 μMol) for 72 hours with or without pre-treated with AT1a antagonist, Valsartan (10 μMol) or pErk inhibitor, PD98.059 (10 μMol) for two hours before stimulation with AngII. After 72 hours of *in vitro* stimulation, RNAs were extracted from B cells using Trizol reagent. In experiments where *Apoe* ^−/−^ */Baffr* ^−/−^ mice were transferred with CD45.1^+^ B cells, the transferred B cells (CD45.1^+^) and the resident B cells (CD45.1^−^) were sorted using flow cytometry after 7 days of AngII infusion and the sorted cells were used for RNA isolation. cDNA was synthesised according to Qiagen reverse Transcription kit. Quantitative real-time PCR (Q-PCR) was performed on an ABI Prism 7700 (Applied Biosystems) in triplicates. CT for *Gapdh* (primers: *Gapdh* R, 5′-CGTCCCGTAGACAAAATGGTGAA-3′; *Gapdh* L, 5′-GCCGTGAGTGGAGTCATACTGGAACA-3′) was used to normalize gene expression. Primer sequences for *Il10* and *Agtr1a* were as follows: *Il-10* Forward: 5′-AAGTGATGCCCCAGGCA-3′, Il10 Reverse: 5′-TCTCACCCAGGGAATTCAAA-3′, *Agtr1a* Forward 5′-AAC AGC TTG GTG GTG ATC GTC-3′, *Agtr1a* Reverse: 5′-CAT AGC GGT ATA GAC AGC CCA-3′.

### Statistics

All data are expressed as mean ± SE. Comparisons of 2 different groups were analyzed by Mann-Whitney *U* test. ANOVA test with Bonferroni’s post-test analysis was used for more than 2 groups. Statistical analyses were performed with Prism 5 software (GraphPad). P values < 0.05 was considered statistically significant.

## Results

### AngII promotes atheroprotective regulatory B cells

**Figure 1:**
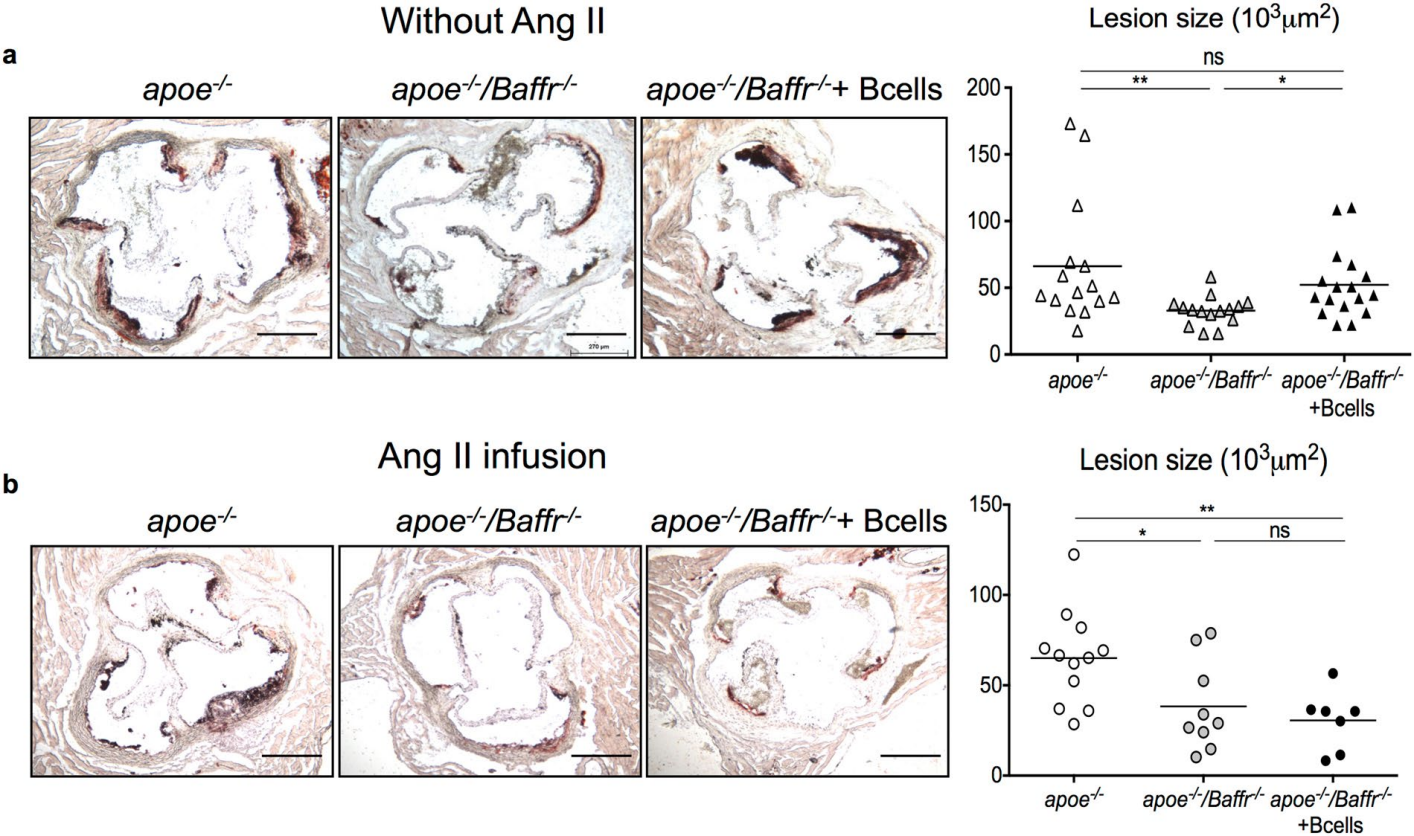
B cell deficiency reduces AngII induced atherosclerosis but adoptive transfer of B cells does not reverse atheroprotection in mice infused with AngII. (**a**) Representative pictures (left), quantitative lesion assessment by Oil Red O staining in the aortic sinus (right) of *Apoe* ^−/−^ and *Apoe* ^−/−^ */Baffr* ^−/−^ mice with or without B cell transfer infused with PBS for 28 days (*Apoe* ^−/−^, n = 15; *Apoe* ^−/−^ */Baffr* ^−/−^, n = 15; *Apoe* ^−/−^ */Baffr* ^−/−^ + B cells, n = 17). PBS was infused in 8-week old mice during 28 days. B cell supplementation was carried out 3 weeks before PBS infusion in one *Apoe* ^−/−^ */Baffr* ^−/−^ group. (**b**) Representative pictures (left) and quantitative lesion assessment by Oil Red O staining in the aortic sinus (right) of *Apoe* ^−/−^ and *Apoe* ^−/−^ */Baffr* ^−/−^ mice transferred with B cells in the presence of AngII (*Apoe* ^−/−^, n = 12; *Apoe* ^−/−^ */Baffr* ^−/−^, n = 9; *Apoe* ^−/−^ */Baffr* ^−/−^ + B cells, n = 7). AngII (1 µg/kg/min) was infused in 8-week old mice during 28 days. B cell supplementation was carried out 3 weeks before AngII infusion in one *Apoe* ^−/−^ */Baffr* ^−/−^ group. *p < 0.05, **p < 0.01, n.s denotes non significance. Scale bar 200 μm.

B2 cells promote experimental atherosclerosis. Here, we aimed to investigate the role of B cells in athero-prone mice infused with AngII, which mimics some clinical situations in patients with coronary artery disease who may display increased ACE activity^27^. As previously reported in mice without AngII infusion^18^, treatment of *Apoe* ^−/−^ mice with anti-CD20 B cell-depleting monoclonal antibody also resulted in a significant reduction of atherosclerosis in mice infused with AngII for 28 days (Supplementary Fig. S1a), with no difference in plasma total cholesterol levels (Supplementary Fig. S1b). Consistent with acute B cell depletion, chronic B2 cell deficiency in *Apoe* ^−/−^ */Baffr* ^−/−^ mice (Supplementary Fig. S2) reduced the development of atherosclerosis compared to *Apoe* ^−/−^ mice, both in the absence and presence of AngII (Fig. 1a,b). Based on these results, we hypothesized that transfer of B cells into *Apoe* ^−/−^ */Baffr* ^−/−^ mice would reverse atheroprotection. We therefore supplemented *Apoe* ^−/−^ */Baffr* ^−/−^ mice with purified splenic CD19^+^IgM^+^ B cells (Supplementary Fig. S3). Replenishment of *Apoe* ^−/−^ */Baffr* ^−/−^ mice with wild-type B cells (WT, C57BL/6J) reversed the atheroprotective effects of B cell deficiency in the absence of AngII infusion, resulting in increased lesion formation (Fig. 1a). These results are consistent with the pro-atherogenic role of Baff receptor signaling in B cells^19^. However, to our surprise, adoptive transfer of B cells did not alter the development of atherosclerosis in *Apoe* ^−/−^ */Baffr* ^−/−^ mice infused with AngII (Fig. 1b). Similar results were obtained when *Apoe* ^−/−^ */Baffr* ^−/−^ mice were replenished with *Apoe* ^−/−^ B cells and infused with AngII (data not shown). Adoptive transfer of B cells in the presence or absence of AngII infusion did not have any effect on plasma total cholesterol levels (Supplementary Fig. S4). Moreover, transfer of B cells did not affect the blood pressure response to AngII (Supplementary Fig. S5). In another series of experiments, we found that AngII infusion was able to increase the development of atherosclerosis in the aortic sinus of *Apoe* ^−/−^ *Baffr* ^−/−^ mice. However, replenishment of *Apoe* ^−/−^ *Baffr* ^−/−^ mice with wild type B cells completely abolished the atherogenic effect of AngII infusion (Supplementary Fig. S6). Thus, our data indicate that the presence of AngII impairs the pro-atherogenic effect of B cells.

Recent studies reported a role for IgM secreting B1a B cells in atheroprotection^20^. Chronic B cell deficiency resulted in a significant reduction of anti-oxLDL IgG and IgM antibodies, and adoptive transfer of B cells restored the levels of both IgG and IgM anti ox-LDL antibodies, independently of AngII infusion (Supplementary Fig. S7). Moreover, we did not find any difference in peritoneal B1a B cell numbers between the various groups of mice (data not shown).

**Figure 2:**
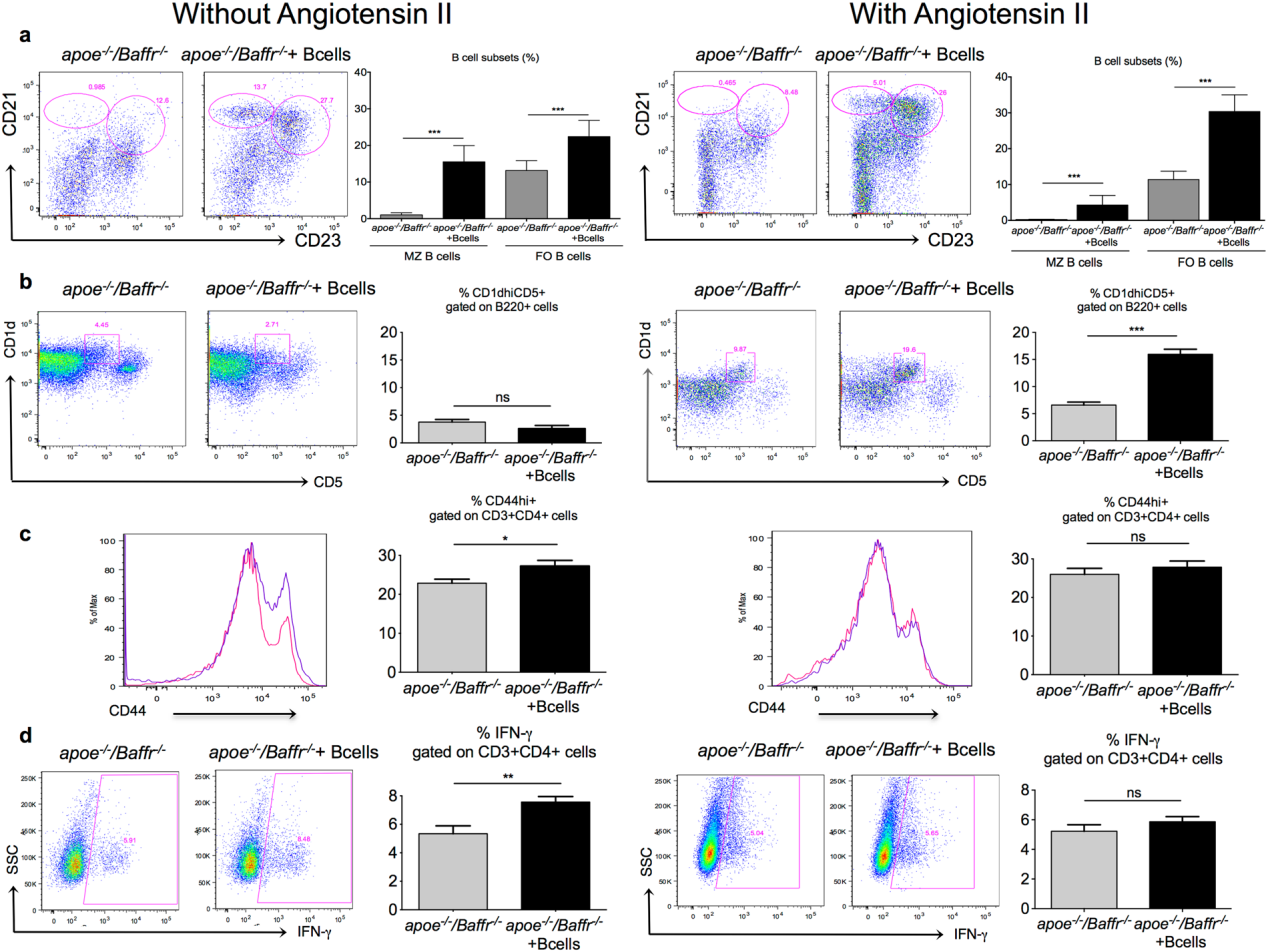
AngII infusion promotes regulatory B cells in B cell replenished *Apoe* ^−/−^ */Baffr* ^−/−^mice. (**a**) Representative dot plots and quantitative analysis of flow cytometry staining of splenic B cell populations. Marginal zone (MZ) B cells are defined as CD21^hi^CD23^−^ population and follicular B cells (FO) are defined as CD21^+^CD23^+^ population amongst B220^+^ cells. Adoptive transfer of *Apoe* ^−/−^ */Baffr* ^−/−^ mice with B cells without (left) or with (right) AngII demonstrates replenishment of all B cell subsets. n = 6–8 mice per group. ***p < 0.001. (**b**) Representative dot plots and quantitative analysis of facs staining of splenic regulatory CD1d^hi^CD5^+^ B cells gated on B220^+^ cells in *Apoe* ^−/−^ */Baffr* ^−/−^ mice with or without adoptive B cell transfer, infused with PBS for 28 days (left) and their age matched controls with AngII infusion (right). (**c**) Representative FACS examples and quantitative analysis of splenic CD44^hi^ cells amongst CD4^+^ T cells in *Apoe* ^−/−^ */Baffr* ^−/−^ mice with or without adoptive B cell transfer, infused with PBS for 28 days (left) and their age matched controls with AngII infusion (right). (**d**) Representative examples and quantitative analysis of splenic IFN-γ^+^ cells amongst CD4^+^ T cells by intracellular facs staining in *Apoe* ^−/−^ */Baffr* ^−/−^ mice with or without adoptive B cell transfer, infused with PBS for 28 days (left) and their age matched controls with AngII infusion (right). n = 6–8 mice per group. *p < 0.05, **p < 0.01, ***p < 0.001, ns denotes non significance.

Our flow cytometry analysis of splenocytes following B cell transfer demonstrated significant replenishment of the major B2 cell subsets (follicular and marginal zone B cells) in *Apoe* ^−/−^ */Baffr* ^−/−^ mice (Figs 2a and S8). Notably however, B cell transfer was associated with a significant increase of CD1d^hi^CD5^+^ regulatory B cell subset only in mice infused with AngII (Figs 2b and S9). Further analysis of the immune response revealed a significant increase of CD4^+^ T cell activation and cytokine (IFN-γ/IL-17/IL-10) production after B cell transfer in the absence of AngII. In contrast, B cell transfer did not lead to any T cell activation in the presence of AngII (Figs 2c,d, S10 and S11). B cell supplementation into *Apoe* ^−/−^ */Baffr* ^−/−^ mice did not significantly affect CD4^+^CD25^high^Foxp3^+^ regulatory T cell population, regardless of AngII infusion (data not shown). Collectively, these results point to an unexpected function of AngII in promoting atheroprotective regulatory B cells. Since the effect is observed in a context of high BAFF levels (*Apoe* ^−/−^ */Baffr* ^−/−^), we suspected a synergistic effect between AngII and BAFF.

### AngII and BAFF synergistically induce IL-10 production in B cells

**Figure 3:**
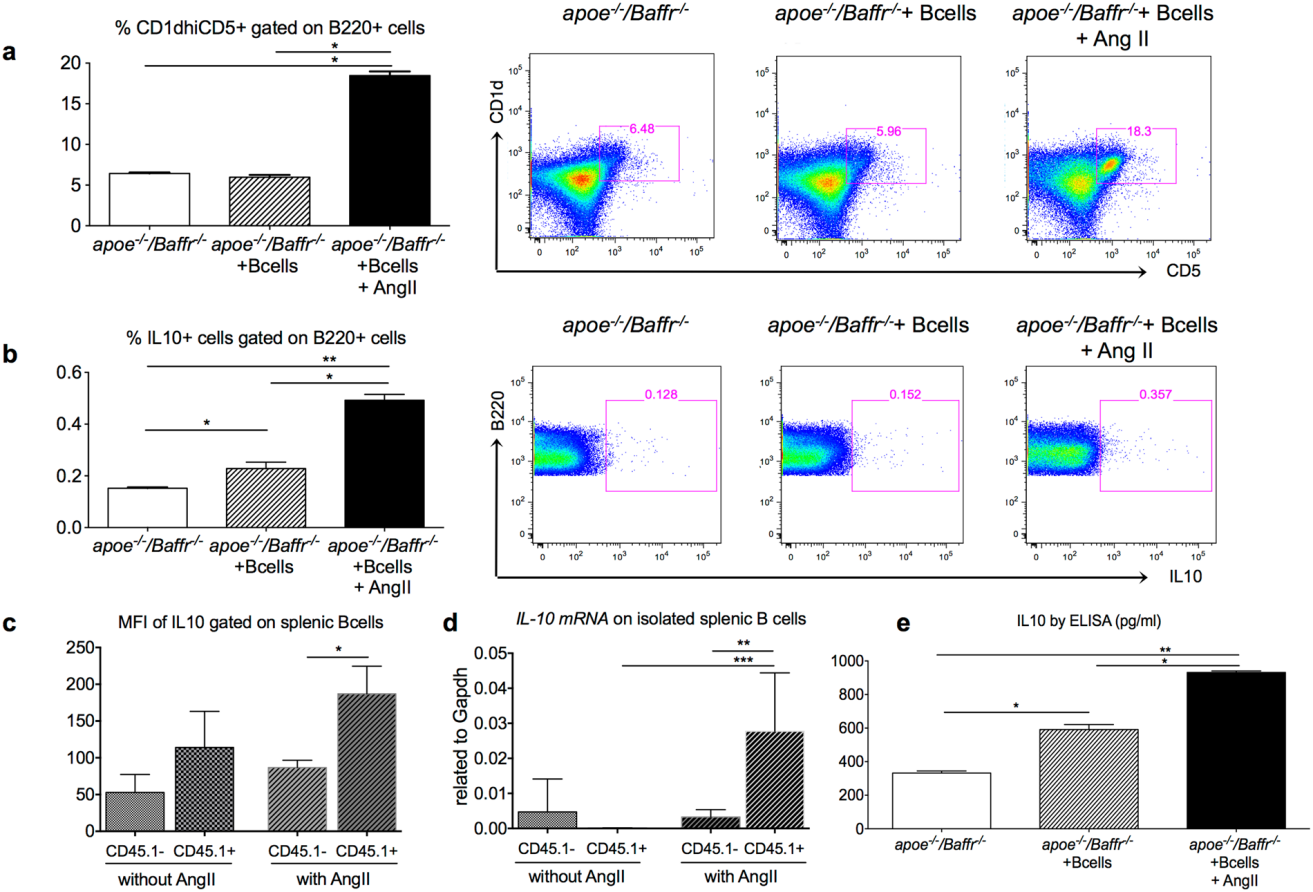
Adoptively transferred B cells in *Apoe* ^−/−^ */Baffr* ^−/−^ mice produced IL10 following AngII infusion. (**a**–**c**) B cells isolated from *Apoe* ^−/−^ */Baffr* ^−/−^ mice without or with AngII infusion for 7 days in the presence or absence of adoptively transferred CD45.1 B cells were stimulated *ex vivo* with anti-CD40/anti-IgM. n = 4 per group. (**a**) Quantitative analysis (left) and representative dot plots (right) of CD1d^hi^CD5^+^ regulatory B cells gated on B220^+^ population shows a significant increase in regulatory B cell population in mice infused with AngII for 7 days. (**b**) Quantitative analysis (left) and representative dot plots (right) of intracellular IL10 staining in B cells gated on B220^+^ population shows a significant increase in B10 cells in mice infused with AngII for 7 days. (**c**) Mean fluorescence intensity of IL10 staining gated on CD19^+^ CD45.1^−^ resident B cells and CD19^+^ CD45.1^+^ transferred B cells from *Apoe* ^−/−^ */Baffr* ^−/−^ mice adoptively transferred with CD45.1 B cells and infused without or with AngII infusion. n = 4–5 per group. (**d**) Quantitative analysis of *Il10 mRNA* expression from resident CD45.1^−^ and transferred CD45.1^+^ B cells isolated from *Apoe* ^−/−^ */Baffr* ^−/−^ mice adoptively transferred with CD45.1 B cells and infused without or with AngII infusion. n = 4–5 per group. (**e**) Splenic B cells isolated from *Apoe* ^−/−^ */Baffr* ^−/−^ mice without or with AngII infusion for 7 days in the presence or absence of adoptively transferred CD45.1 B cells were stimulated *ex vivo* with LPS to detect soluble IL10 levels by ELISA. n = 4 per group. *p < 0.05, **p < 0.01, ****p < 0.001.

Next, we investigated if the increase of CD1d^hi^CD5^+^ B cells in *Apoe* ^−/−^ */Baffr* ^−/−^ mice following AngII infusion was due to an expansion of endogenous immature residual B cells or to the population of adoptively transferred B cells being polarized toward a regulatory phenotype. To answer this question, we transferred CD45.1 splenic B cells into *Apoe* ^−/−^ */Baffr* ^−/−^ mice with or without AngII infusion for 7 days. We found a significant increase of CD1d^hi^CD5^+^ B cells in the AngII infused group (Fig. 3a). Consistent with a regulatory CD1d^hi^CD5^+^ phenotype, AngII also induced IL-10 production in B220^+^/CD19^+^ B cells (Figs 3b and S12, and data not shown), and CD1d^hi^CD5^+^ B cells (Supplementary Fig. S13), but not in B1 cells (Supplementary Fig. S14). Amongst the transferred CD45.1^+^ B cells in *Apoe* ^−/−^ */Baffr* ^−/−^ mice, IL-10 levels were significantly higher in the mice infused with AngII (Fig. 3c), suggesting that the transferred mature B cells produced IL-10 upon AngII stimulation. Our results were verified by qPCR (Figs 3d and S15) and by ELISA (Fig. 3e).

Interestingly, we also observed a significant increase of IL-10 production in *Apoe* ^−/−^ */Baffr* ^−/−^ mice transferred with B cells in the absence of AngII, although to a lower extent compared with AngII-treated mice (Figs 3b, S12 and S16). In fact, *Baffr* deficiency results in a significant increase of serum BAFF levels (Supplementary Fig. S17) and recent studies demonstrated that both murine and human B cells can be induced to produce IL-10 upon stimulation with BAFF^33, 34^. We therefore addressed the potential synergy between AngII and BAFF in promoting a B10 phenotype. We transferred B cells to either *Apoe* ^−/−^ mice (‘normal’ plasma BAFF levels) or *Apoe* ^−/−^ */Baffr* ^−/−^ mice (elevated BAFF levels), and treated the mice with PBS or AngII for 7 days. Our flow cytometry analysis of splenocytes from these mice showed a significant increase of IL-10 production by B cells, especially in B cell replenished *Apoe* ^−/−^ */Baffr* ^−/−^ mice with AngII infusion (Supplementary Fig. S12b). Our ELISA results showed that there was a significant increase of IL-10 production in AngII infused *Apoe* ^−/−^ mice; however, IL-10 production was more pronounced in *Apoe* ^−/−^ */Baffr* ^−/−^ mice infused with AngII (Supplementary Fig. S16), revealing a synergy between AngII and BAFF in the induction of B10 cells.

### Regulatory B cells generated in response to AngII are atheroprotective through their production of IL-10

**Figure 4:**
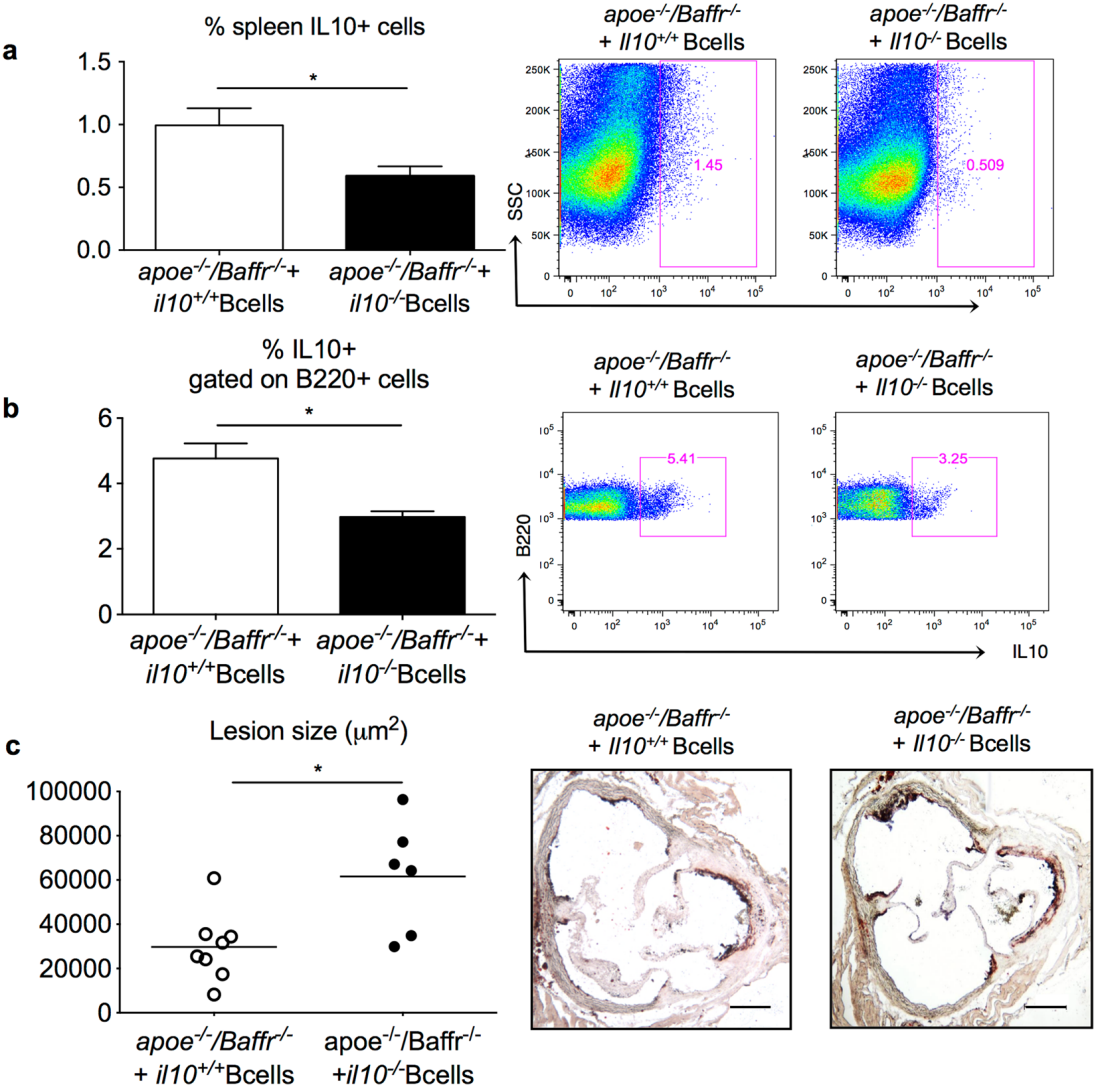
Regulatory B cells generated in response to AngII are atheroprotective through their production of IL-10. *Apoe* ^−/−^ */Baffr* ^−/−^ mice were replenished with *Il10* ^+/+^ or *Il10* ^−/−^ B cells and infused with AngII for 28 days. (**a**) Quantitative analysis (left) and representative dot plots (right) of intracellular staining of IL10 in total splenocytes demonstrates a significant reduction of IL10 levels in mice transferred with *Il10* ^−/−^ B cells. n = 6 mice per group. (**b**) Quantitative analysis (left) and representative dot plots (right) of intracellular staining of IL10 in splenic B cells demonstrates a significant reduction of B10 in mice transferred with *Il10* ^−/−^ B cells. n = 6 mice per group. (**c**) Quantitative lesion assessment by Oil Red O staining in the aortic sinus (left) and representative pictures (right) of *Apoe* ^−/−^ */Baffr* ^−/−^ mice transferred with *Il10* ^+/+^ or *Il10* ^−/−^ B cells and infused with AngII for 28 days (n = 6–8 mice per group). *p < 0.05. Scale bar 200 μm.

We hypothesized that AngII-induced regulatory B cells prevented the progression of atherosclerosis through their IL-10 production. To test this hypothesis, we re-supplemented *Apoe* ^−/−^ */Baffr* ^−/−^ mice with either *Il-10* ^+/+^ or *Il-10* ^−/−^ B cells, followed by AngII infusion for 28 days. Adoptive transfer of *Apoe* ^−/−^ */Baffr* ^−/−^ mice with *Il-10* ^−/−^ B cells did not alter BAFF levels (Supplementary Fig. S17b) but resulted in a significant reduction of IL-10 production in splenocytes (Fig. 4a), splenic B cells (Figs 4b and S18) and CD1d^hi^CD5^+^ B cells (data not shown), and was associated with a significant acceleration of atherosclerosis (Fig. 4c). These results demonstrate that AngII induced B10 cells are endowed with atheroprotective properties.

### *Agtr1a* deficiency in B cells abrogates the atheroprotective B10 phenotype

**Figure 5:**
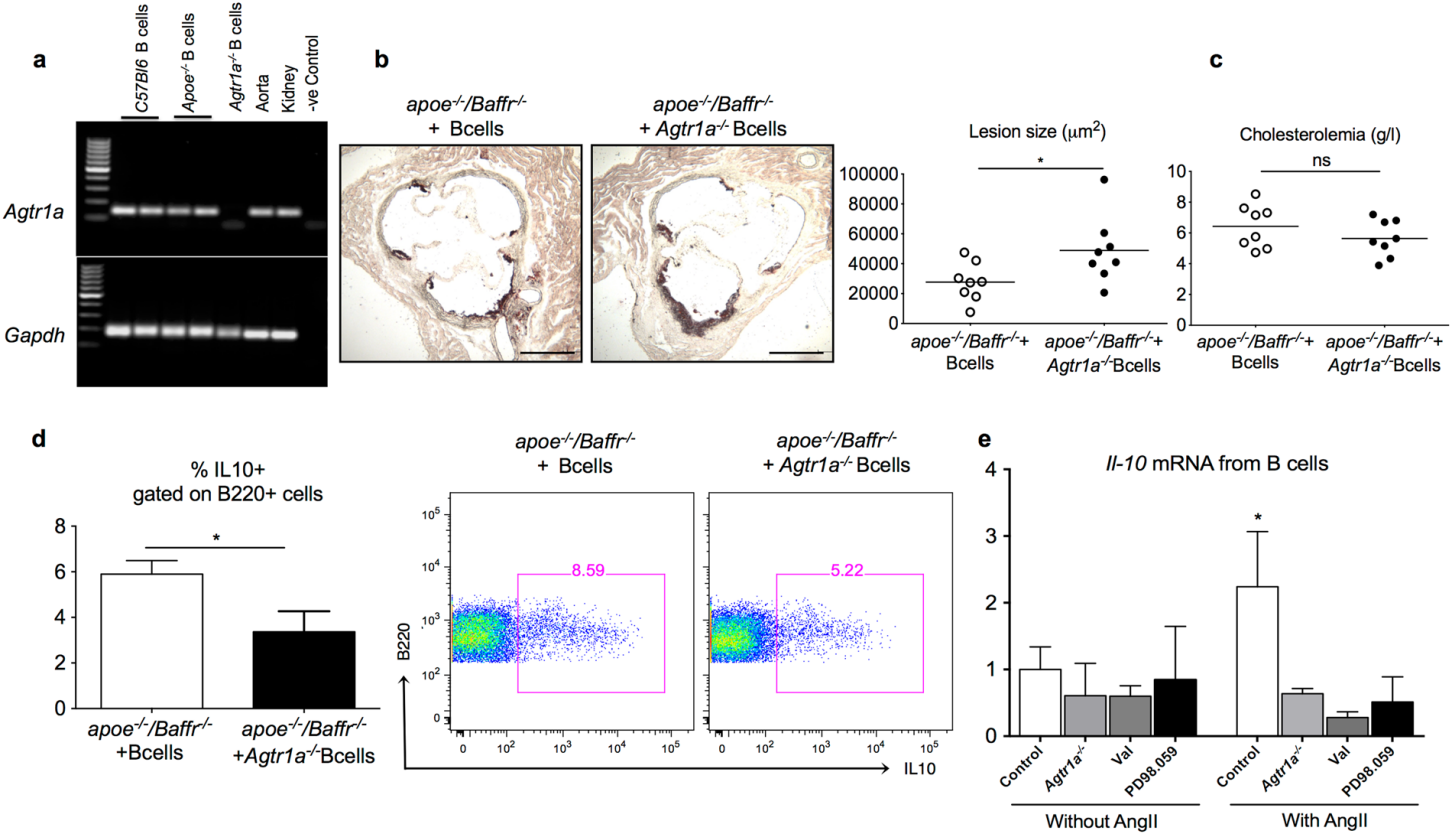
Agtr1a deficiency in B cells accelerates AngII induced atherosclerosis in B cell replenished *Apoe* ^−/−^ */Baffr* ^−/−^ mice. (**a**) Agarose gel picture depicting *Agtr1a* gene expression in C57BL/6J WT, *Apoe* ^−/−^ and *Apoe* ^−/−^ */Baffr* ^−/−^ B cells and lack of expression in *Agtr1a* ^−/−^ B cells. Aorta and kidney samples were used as positive control. (**b**) Representative pictures (left) and quantitative lesion assessment by Oil Red O staining in the aortic sinus (right) and plasma cholesterol levels (**c**) of *Apoe* ^−/−^ */Baffr* ^−/−^ mice replenished with *Agtr1a* ^+/+^ or *Agtr1a* ^−/−^ B cells and infused with AngII for 28 days. n = 8 mice per group. (**d**) Quantitative analysis (left) and representative dot plots (right) of intracellular IL10 staining gated on splenic B220^+^ cells of *Apoe* ^−/−^ */Baffr* ^−/−^ mice replenished with *Agtr1a* ^+/+^ or *Agtr1a* ^−/−^ B cells and infused with AngII for 28 days. n = 8 mice per group. (**e**) Quantitative measurements of *Il10* mRNA in isolated C57Bl6 or *Agtr1a* ^−/−^ B cells treated *in vitro* in the presence or absence of AngII with or without pre-treatment with valsartan or PD98.059, antagonists of Agtr1a and ERK pathway, respectively. N = 4–6 per treatment condition and pooled data from 2 independent experiments. *p < 0.05, ns denotes non significance. Scale bar 200 μm.

Agtr1a is known to mediate several biological effects of AngII^35, 36^. In accordance with a previous publication^10^, we observed *Agtr1a* gene expression in splenic purified B cells (Fig. 5a). We therefore investigated if the AngII-induced atheroprotective effects of B cells were mediated by Agtr1a. Adoptive transfer of *Agtr1a* ^−/−^ B cells into AngII infused *Apoe* ^−/−^ */Baffr* ^−/−^ mice abrogated the atheroprotection observed in *Apoe* ^−/−^ */Baffr* ^−/−^ mice transferred with WT B cells, despite no changes in plasma total cholesterol levels (Fig. 5b,c). We also observed a significant 38% reduction of IL-10 production by B cells (Figs 5d, S19 and S20) in mice transferred with *Agtr1a* ^−/−^ B cells. *In vitro* stimulation of WT B cells with AngII induced *Il-10 mRNA*, which was abrogated by *Agtr1a* deficiency and by valsartan, an antagonist of Agtr1a (Fig. 5e). We further attempted to understand the pathway through which Agtr1a induces IL-10 production. ERK activation is critical for the induction of IL-10 in many cell types^37^ and AngII is known to induce the ERK pathway^38, 39^. Accordingly, ERK inhibition using PD98.059 abolished AngII induced *Il-10* mRNA expression in B cells (Fig. 5e). Our results identify a previously unsuspected role for AngII in inducing IL-10 production by B cells through Agtr1a-dependent activation of ERK.

## Discussion

The present study reveals a synergistic effect between AngII and BAFF in inducing atheroprotective B10 cells via AT1A receptor. Our conclusion is supported by four major findings. First, adoptive transfer of B cells in *Apoe* ^−/−^ */Baffr* ^−/−^ mice reversed the atheroprotective effect of B cell deficiency only in the absence of AngII infusion. Second, AngII infusion led to a profound increase of the regulatory CD1d^hi^CD5^+^ B10 subset and its production of IL-10. Third, replenishment of *Apoe* ^−/−^ */Baffr* ^−/−^ mice with *Il-10* ^−/−^ B cells broke the resistance to atherosclerosis observed in AngII-infused *Apoe* ^−/−^ */Baffr* ^−/−^ mice replenished with *Il-10* ^+/+^ B cells. Finally, AngII induced IL-10 production by B cells was significantly attenuated by Agtr1a deficiency, and transfer of *Apoe* ^−/−^ */Baffr* ^−/−^ mice with *Agtr1a* ^−/−^ B cells in the presence of AngII led to increased atherosclerosis compared with the transfer of *Agtr1a* ^+/+^ B cells.

Consistent with previous reports on the pro-atherogenic role of BAFF receptor signaling in B cells^18, 19, 21^, we observed that replenishment of *Apoe* ^−/−^ */Baffr* ^−/−^ mice with mature B cells significantly promoted atherosclerosis. However, to our surprise replenishment of *Apoe* ^−/−^ */Baffr* ^−/−^ mice with mature B cells in did not reverse atheroprotection when mice were infused with AngII. Consistent with this finding, AngII infusion increased atherosclerosis in the aortic sinus of *Apoe* ^−/−^ */Baffr* ^−/−^ mice but AngII pro-atherogenic effect was lost in mice replenished with mature B cells. Recent published studies have shown differential roles of B cell subsets in the development of atherosclerosis. While mature B2 cells are shown to be proatherogenic, the IgM secreting B1a subset is shown to be atheroprotective^20, 22^. The latter subset was unlikely to be involved in the AngII-dependent atheroprotective B cell phenotype because restoration of (anti-oxLDL) IgM antibody levels following B cell transfer in *Apoe* ^−/−^ */Baffr* ^−/−^ mice was comparable in mice with or without AngII. Though adoptive transfer of mature B cells in *Apoe* ^−/−^ */Baffr* ^−/−^ mice led to similar restoration of B cell subsets in presence or absence of AngII, we observed a profound increase of CD1d^hi^CD5^+^ regulatory B cell subset only in mice infused with AngII. This was associated with a reduction in CD69 and CD44high expression by CD4+ T cells, suggesting that the expanded regulatory B cell population (under AngII) inhibited CD4+ T cell activation. Our data underlines the tight cooperation between adaptive T and B cell subsets^40^ in regulating atherogenic immune responses.

We identified IL-10 production by B cells as responsible for the atheroprotective phenotype of AngII-induced regulatory B cells, and revealed a requirement for both AngII and BAFF in promoting atheroprotective B10 cells. Therefore, our data indicate that B10 cells control atherosclerosis under defined conditions. There are several clinical settings where those conditions are met and may promote the generation of disease modulating B10 cells. It is noteworthy that in patients with active chronic sarcoidosis, elevated levels of BAFF and ACE were strongly associated with increased IL-10 producing B cells^26, 41^. CD20 mAb therapy in humans is known to increase BAFF levels and the naïve (CD19^+^IgD^+^), transitional or plasmablast (CD19^+^IgD^+^CD24^hi^CD38^hi^) B cells that recover after B cell depletion are responsive to BAFF and are associated with a better therapeutic effect^42, 43^. Interestingly, regulatory B cells in humans are identified as CD19^+^CD24^hi^CD38^hi^ ^44^. In mice, additional membrane markers of regulatory B cells, non-investigated in our study, have been identified including TIM-1^45^ and CD9^46^. It is plausible that the B cells that regenerate after B cell depletion therapy acquire a regulatory phenotype due to high BAFF levels. Our results suggest that patients who undergo B cell depletion therapy would be protected from atherosclerosis and the B cells that regenerate in the presence of high BAFF and AngII (patients with atherosclerosis have high ACE activity) will enhance the protective effects due to their improved regulatory functions. Recently, Jackson *et al*. have reported another potential mechanism of atheroprotection induced by BAFF. Using several genetically modified mouse models on *Apoe* ^−/−^ background, they showed that BAFF overexpression increased anti-oxLDL IgM antibody plasma levels through TACI activation^47^. However, the functional effect of those B cell subsets in humans is yet to be explored. Increased BAFF level is associated with various autoimmune diseases and neutralizing BAFF is considered to be a potential therapeutic target for many of these diseases^48^. Our results show that therapeutic strategies aimed at neutralizing BAFF should be considered with caution since BAFF also downregulates immune responses through induction of IL-10. All our results were obtained in a BAFF receptor deficient environment and it is important to confirm them in other settings. In addition, as our study was performed in mice at an early stage of atherosclerosis, further studies are required to confirm our findings in more advanced atherosclerosis.

Recently, Chan *et al*. reported a reduction of AngII-induced hypertension in *Apoe* ^−/−^ */Baffr* ^−/−^ mice compared with control *Apoe* ^−/−^ */Baffr* ^+/+^ mice. In addition, B cell supplementation in *Apoe* ^−/−^ */Baffr* ^−/−^ mice worsened hypertension^49^. In our study, we found a slight but non-significant trend toward a lower increase of blood pressure in *Apoe* ^−/−^ */Baffr* ^−/−^ mice compared to *Apoe* ^−/−^ mice after infusion with AngII. B cell transfer into *Apoe* ^−/−^ */Baffr* ^−/−^ mice did not affect the blood pressure response to AngII infusion. This apparent discrepancy between our results and those of Chan *et al*. with regard to the hypertensive phenotype could be explained by the different doses of AngII used in the 2 studies (1 μg/kg/min or 1.4 mg/kg/day versus 0.7 mg/kg/day).

AngII infusion accelerates atherosclerosis and blockade of the renin-angiotensin system is protective. However, the proatherogenic effects of AngII have been shown to depend on age, sex, AngII dose, cholesterolemia, duration of treatment and the arterial site at which lesion size was assessed^50–52^. Weiss *et al*. have shown that after 4 weeks of Ang II infusion, the increase of atherosclerotic plaque size was modest in the ascending thoracic aorta and was much more pronounced in the descending thoracic and abdominal aorta^53^. Similarly, Zhou *et al*. found that the proatherogenic effect of AngII was much more important in the descending aorta than in the aortic sinus^54^. Lesion size was not statistically different between mice infused with PBS as compared to mice infused with AngII in Fig. 1a,b. However, experiments with and without AngII were not performed at the same time and should not be compared. When experiments with and without AngII were conducted simultaneously in immunodeficient *apoe* ^−/−^ *Baffr* ^−/−^ mice, we confirmed that AngII accelerated atherosclerosis in the aortic sinus. Interestingly, supplemention of *apoe* ^−/−^ *Baffr* ^−/−^ mice with B cells abrogated the pro-atherogenic effect of AngII due to expansion of an IL-10-producing B cell population. Together, the data suggest differential effects of AngII depending on the target cells, being pro-atherogenic in vascular cells and anti-atherogenic in immune cells, like B cells. Previous experiments using bone marrow transplantation in *Ldlr* ^−/−^ mice are in agreement with our findings. Cassis *et al*. found that AngII type 1A receptor engagement on vascular cells clearly promoted atherosclerosis, but the role of AngII type 1A receptor expressed by hematopoietic cells remained unclear^14^. This could be accounted for by the differential effects of AngII type 1A receptor engagement on different leukocyte subsets. For example, AngII may activate Th1 T cells^10^ but AngII type 1A receptor also limits M1 macrophage polarization in part through a reduction of IL1 receptor activation^15^. Thus, the function of AT1A may not be solely proinflammatory. In our study, we found that AngII type 1A receptor activation induced a B10 regulatory profile. One of the pathways via which AngII mediates its cellular effects involves the activation of ERK downstream of AT1A^38, 39^. Furthermore, ERK activation is the common pathway utilized by most immune cells to induce IL-10 production^37^. Our findings extend those results and provide evidence for an ERK-dependent induction of IL-10 production by B cells in response to activation of AT1A receptor.

In summary, our results reveal a previously unsuspected synergistic role between AngII and BAFF in inducing atheroprotective B10 cells. Exploiting those pathways may lead to novel therapeutic strategies to limit atherosclerosis in humans.

## Electronic supplementary material

Supplemental figures 1–20
