# Supplementary material for: Angiotensin II synergizes with BAFF to promote atheroprotective regulatory B cells
Source: Sci Rep. 2017 Jun 23;7:4111. doi: 10.1038/s41598-017-04438-6 (PMC5482806; doi:10.1038/s41598-017-04438-6)
Supplement: Supplementary file 1 — Supplemental figures 1–20 [file 41598_2017_4438_MOESM1_ESM.pdf]

Angiotensin II synergizes with BAFF to promote atheroprotective regulatory B cells

Padmapriya Ponnuswamy, Jeremie Joffre, Olivier Herbin, Bruno Esposito, Ludivine Laurans, Christoph J. Binder, Thomas F. Tedder, Lynda Zeboudj, Xavier Loyer, Andreas Giraud, Yujiao Zhang, Alain Tedgui, Ziad Mallat, Hafid Ait-Oufella

Supplementary Fig. 1

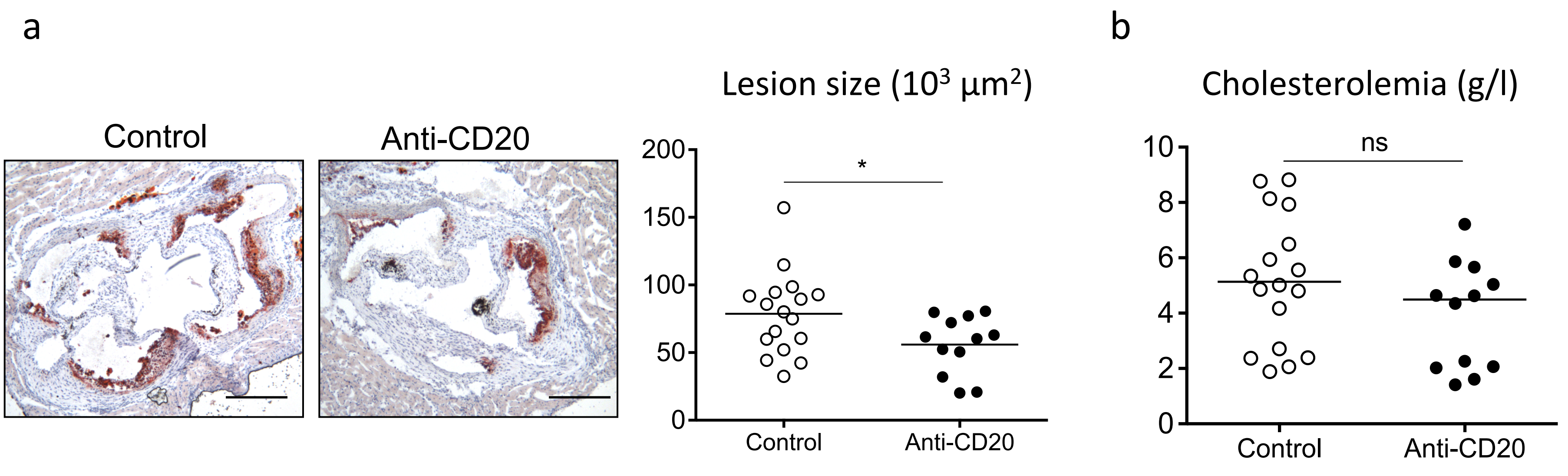

8-week old *apoe$^{-/-}$* mice were infused with AngII (1µg/kg/min) during 28 days and fed a chow diet. One group was treated with anti-CD20 depleting antibody (200µg/14 days) and one control group received isotype control (200µg/14 days). Under AngII infusion, mature B cell depletion reduced significantly atherosclerosis development int the aortic sinus (a) with no difference of plasma cholesterol levels (b). N=12-17/group. NS for non significant, *, $P<0.05$

Supplementary Fig. 2

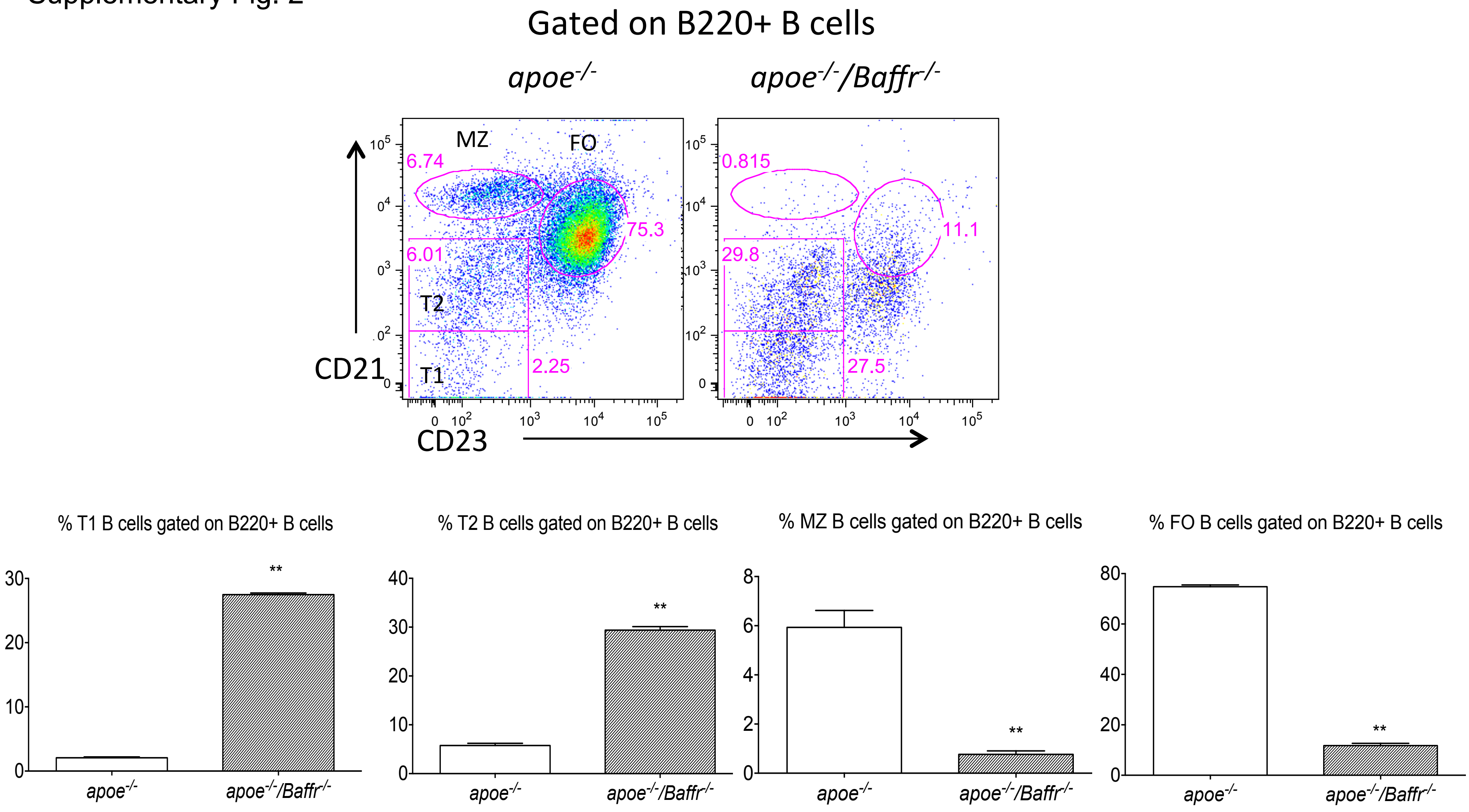

Representative dot plots and quantitative analysis of flow cytometry staining of splenic B cells subsets in *apoe*$^{-/-}$(white) mice and their age matched controls *apoe*$^{-/-}$/*Baffr*$^{-/-}$ (Grey). B cell subsets are expressed as percentage of total B220+ B cells. Marginal zone B cells (MZ) are identified as B220+CD21highCD23-, Follicular B cells (FO) as B220+CD21+CD23+, transitional T1 B cells as B220+CD21-CD23- and transitional T2 B cells are B220+CD21lowCD23-; N=4/group. **p<0.01.

Supplementary Fig. 3

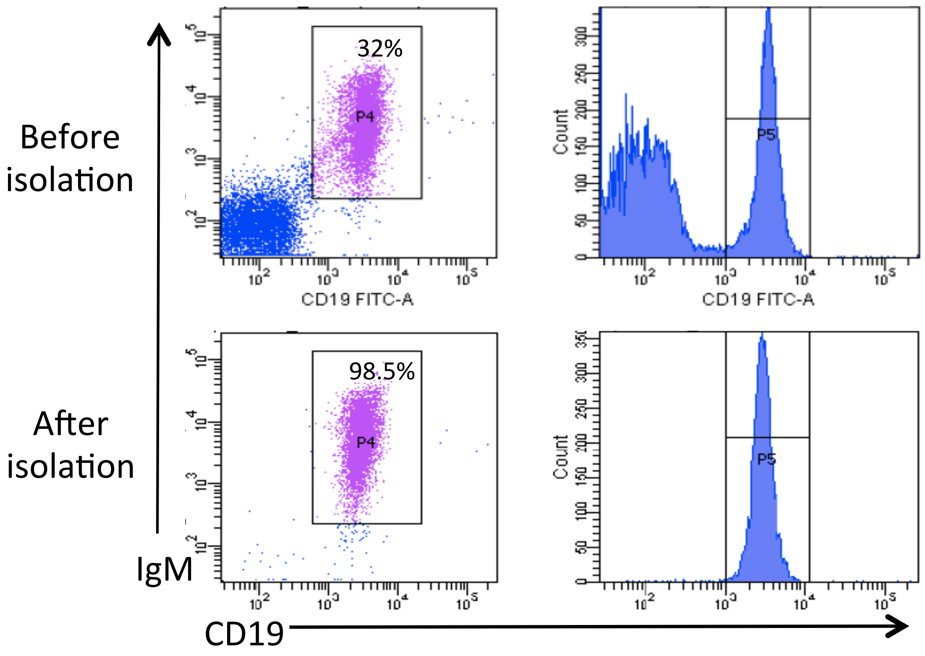

Mature B cells were isolated from apoe$^{-/-}$ spleen using magnetic microbeads. After isolation, purity of IgM+CD19+ B cells was higher than 98%.

Supplementary Fig. 4

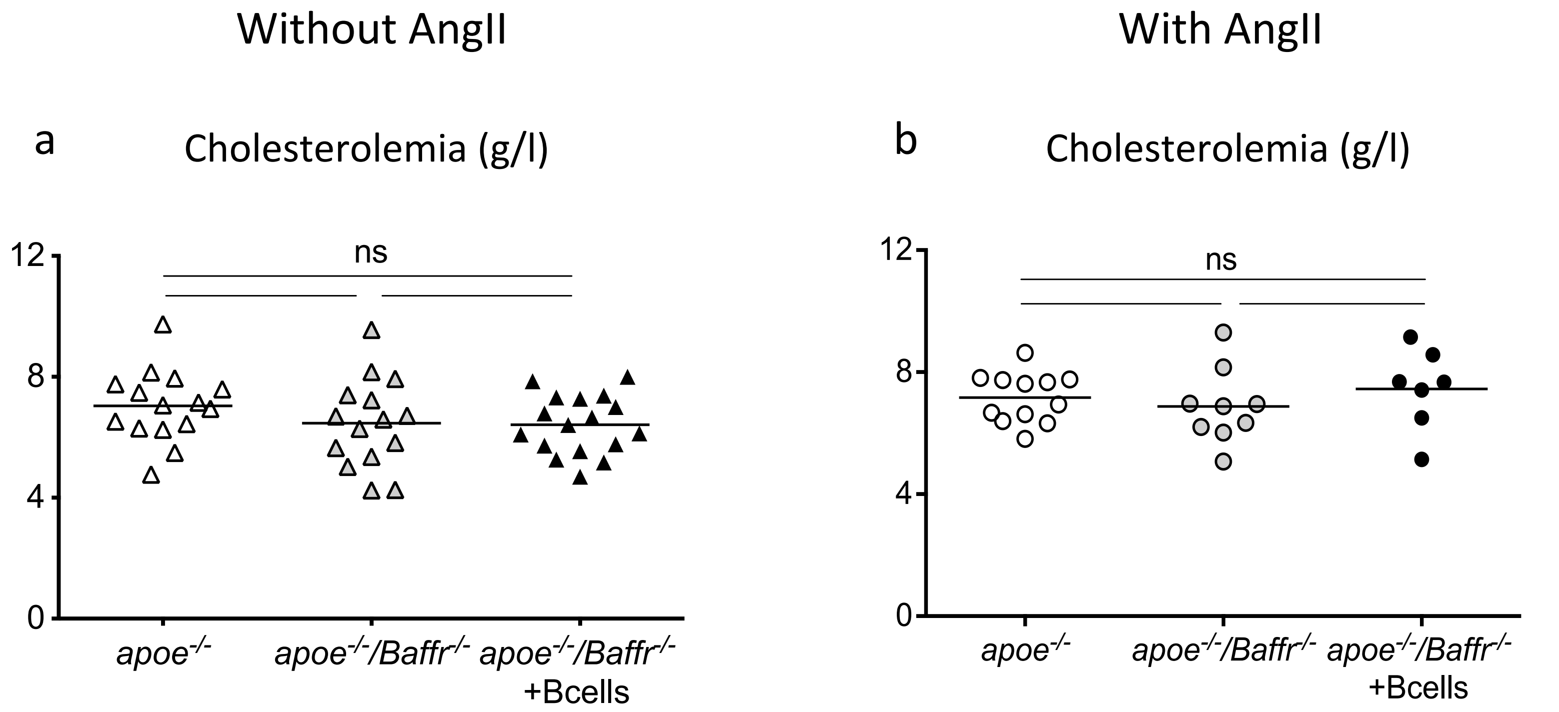

8-week old *apoe*$^{-/-}$ and *apoe*$^{-/-}$*/Baffr*$^{-/-}$ mice were infused with PBS (a) or AngII (1µg/kg/min) (b) during 28 days and fed a chow diet. Supplementation with 30.10^6 mature B cells was done at 5 weeks of age. Plasma cholesterol levels was measured at sacrifice at 12 weeks. We did not observe any difference of cholesterolemia between groups. NS for non significant, AngII for Angiotensin II

Supplementary Fig. 5

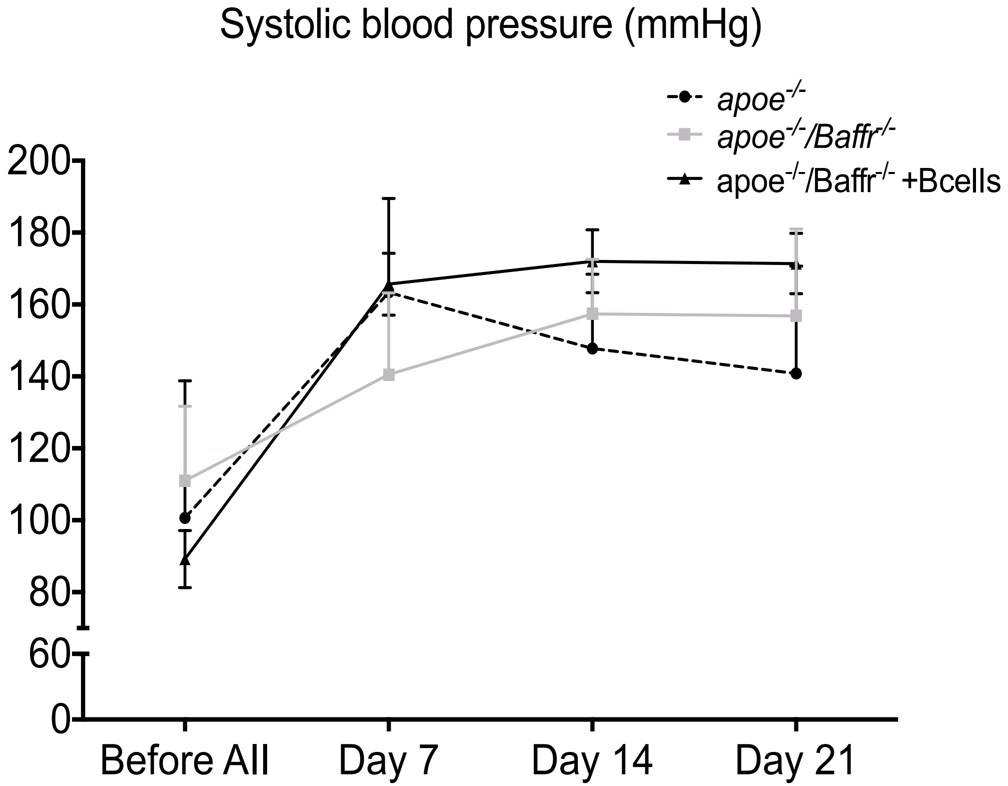

Systolic blood pressure of *apoe*$^{-/-}$, *apoe*$^{-/-}$*/Baffr*$^{-/-}$ *and apoe*$^{-/-}$*/Baffr*$^{-/-}$ mice replenished with B cells followed by AngII infusion (1µg/kg/min) for 28 days. B cell supplementation was done at 5 weeks and AngII infusion was started at 8 weeks. N=8-9/group.

Supplementary Fig. 6

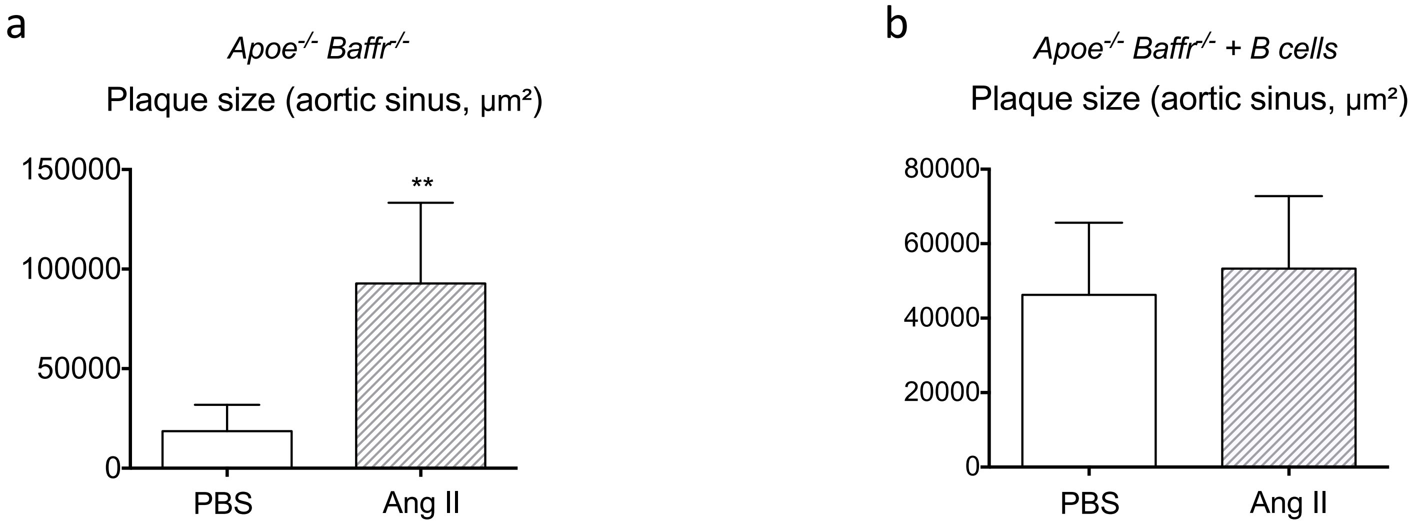

(a) Ten-week old Apoe$^{-/-}$/Baffr$^{-/-}$ mice were infused with PBS or AngII (1µg/kg/min) during 28 days and sacrified. AngII significantly accelerated atherosclerosis in the aortic sinus. N=4-5/group. **P<0.01.

(b) Seven-week old Apoe$^{-/-}$/Baffr$^{-/-}$ mice were resplenished with purified 30.10^{6} purified B cells. 3 weeks later, mice were infused with PBS or AngII (1µg/kg/min) during 28 days and sacrified. AngII had no effect on atherosclerotic lesion size. N=4-5/group.

Supplementary Fig. 7

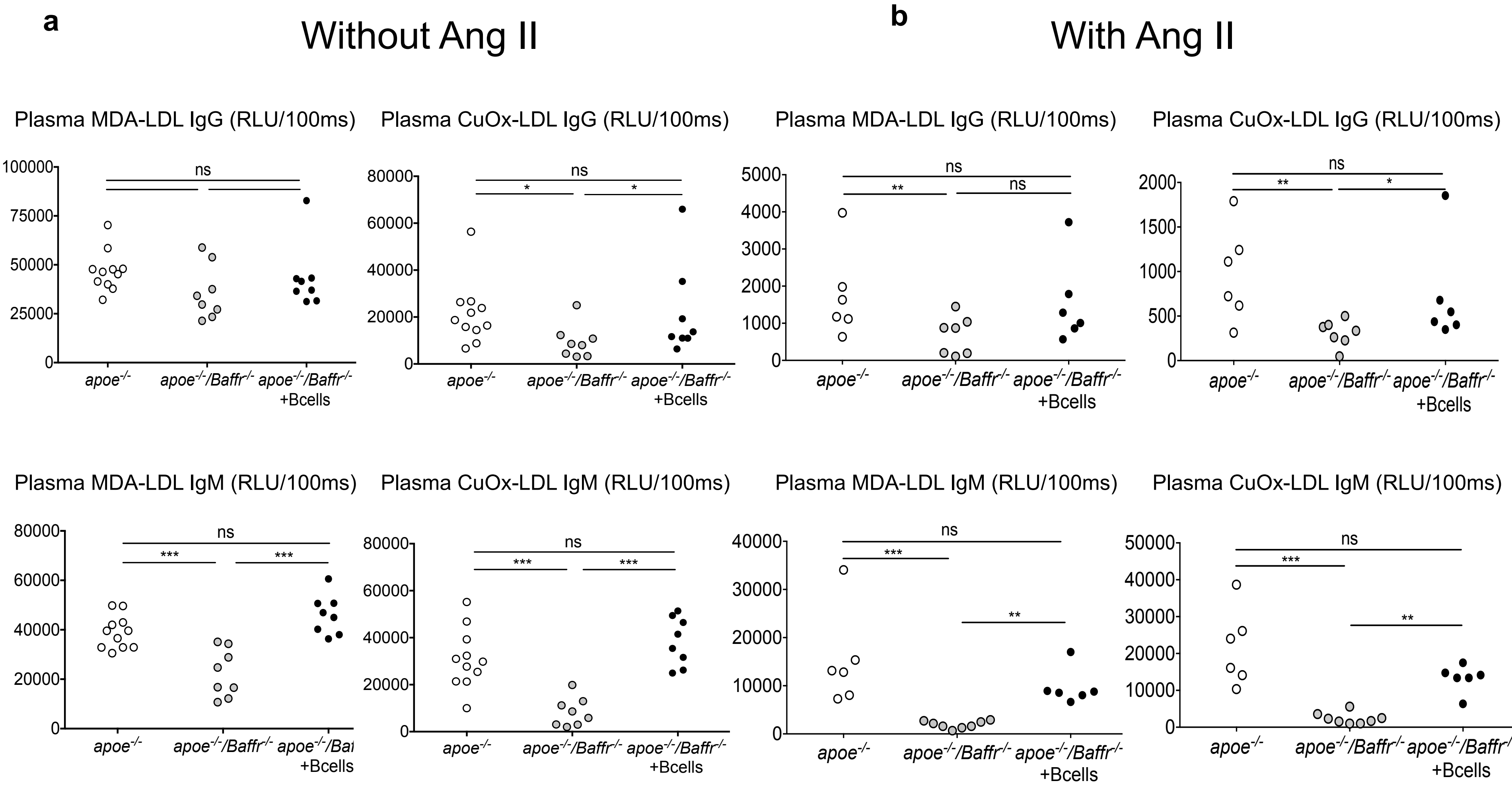

Plasma levels of MDA-LDL and CuOx-LDL IgG and IgM antibodies were assessed by ELISA in *apoe*$^{-/-}$, *apoe*$^{-/-}$/*Baffr*$^{-/-}$ and *apoe*$^{-/-}$/*Baffr*$^{-/-}$ mice replenished with B cells without (a) or with AngII (b) infusion for 28 days. N=8-11 mice per group without AngII infusion and n=6-7 mice per group with AngII infusion. Reduced number of mice in the AngII group is due to mortality increased following AngII infusion. *p<0.05, **p<0.01,***p<0.001 and ns denotes non significance.

Supplementary Fig. 8

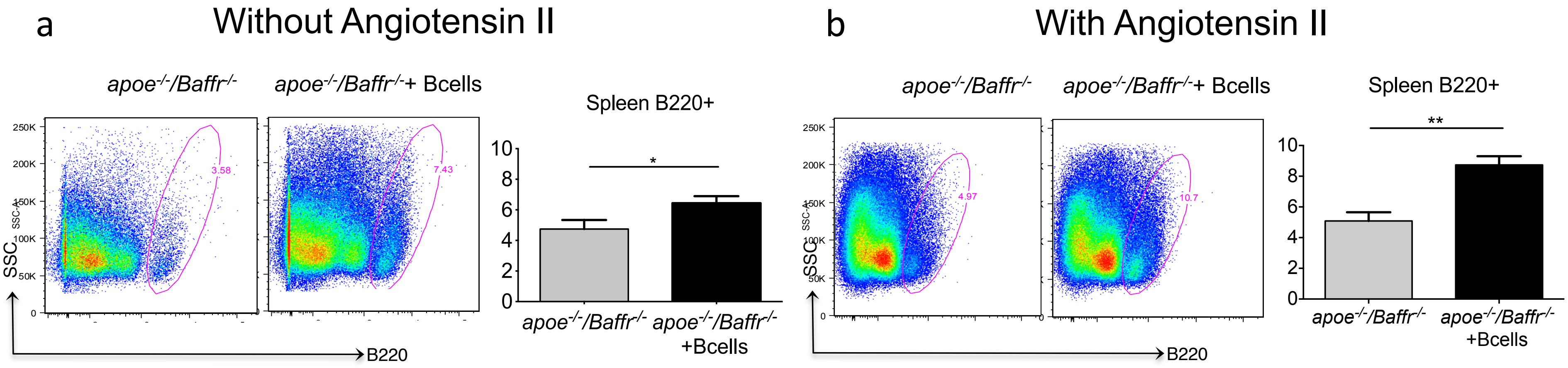

Representative dot plots and quantitative analysis of flow cytometry staining of splenic B220+ B cells in *apoe*$^{-/-}$/*Baffr*$^{-/-}$ mice with or without adoptive B cell transfer, infused without AngII (a) or with AngII for 28 days (b). *p<0.05, **p<0.01. n=8-11 mice per group without AngII infusion and n=7-8 mice per group with AngII infusion.

Supplementary Fig. 9

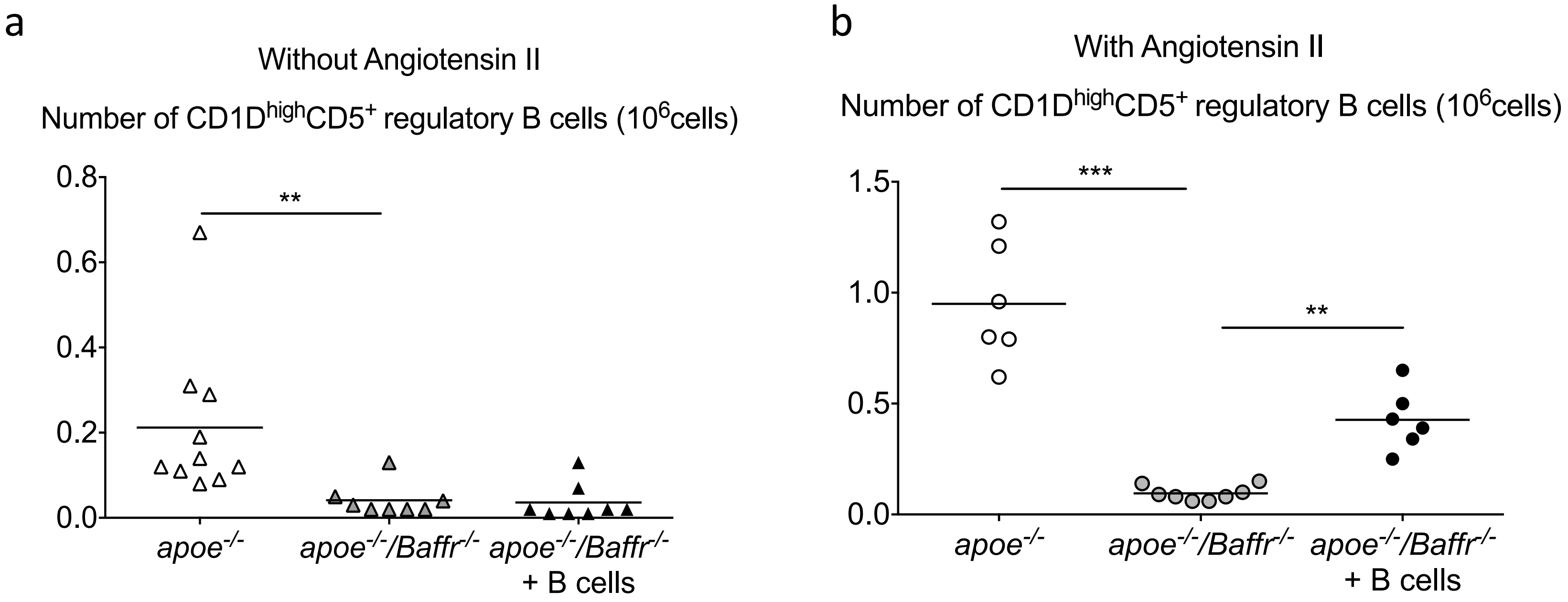

Quantification of splenic Bregs absolute number in experiments without (a) and with (b) angiotensin II infusion (right) at day 28 following PBS or AngII infusion. $**p<0.01$.

Supplementary Fig. 10

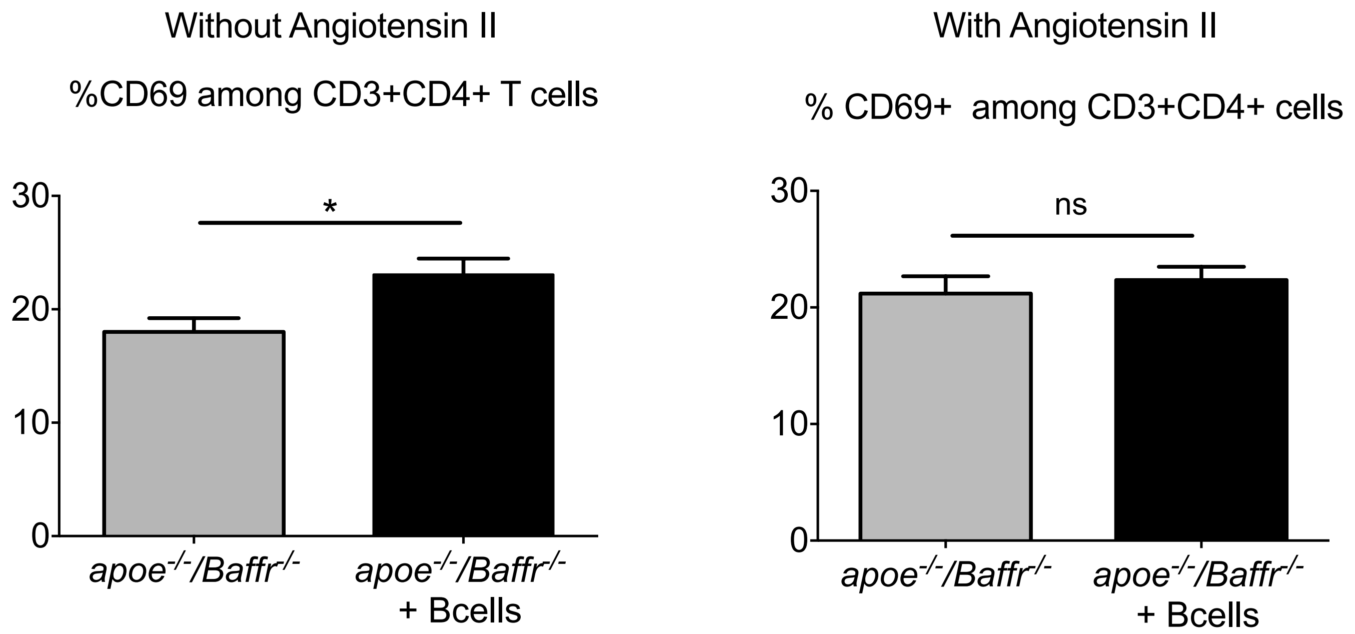

CD69 expression by splenic CD4+ T cells analyzed by flow cytometry after intracellular staining. 8-week old *apoe*$^{-/-}$/*Baffr*$^{-/-}$ mice were infused with PBS or AngII (1µg/kg/min) during 28 days and sacrified. Supplementation with 30.10^6 mature B cells was done 3 weeks before PBS/AngII infusion. Without AngII infusion, B cell supplementation in *apoe*$^{-/-}$/*Baffr*$^{-/-}$ increased CD69 expression by CD4+ T cells. In contrast, in the presence of AngII, B cell transfer did not modify CD69 expression by CD4+ T cells. *, $P<0.05$. n=8-11 mice per group without AngII infusion and n=7-8 mice per group with AngII infusion

Supplementary Fig. 11

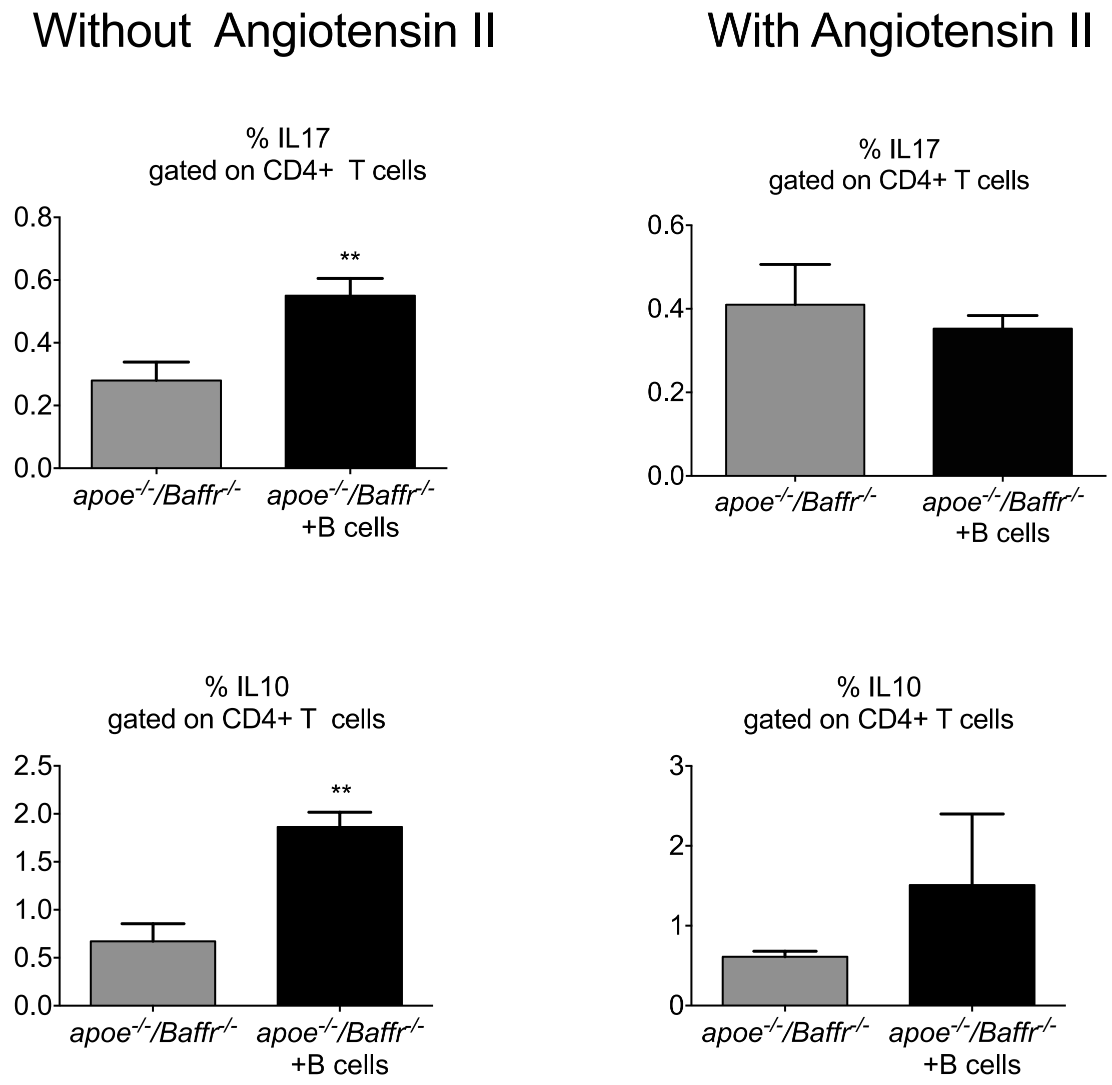

Cytokine production by splenic CD4+ T cells analyzed by flow cytometry after intracellular staining. 8-week old *apoe*$^{-/-}$/*Baffr*$^{-/-}$ mice were infused with PBS or AngII (1µg/kg/min) during 28 days and sacrified. Supplementation with 30.10^6 mature B cells was done 3 weeks before PBS/AngII infusion. Without AngII infusion, B cell supplementation in *apoe*$^{-/-}$/*Baffr*$^{-/-}$ increased IL-17 and IL-10 production by CD4+ T cells. In contrast, in the presence of AngII, B cell transfer did not modify cytokine production by CD4+ T cells. **, $P<0.01$. n=8-11 mice per group without AngII infusion and n=7-8 mice per group with AngII infusion

Supplementary Fig. 12

a

b

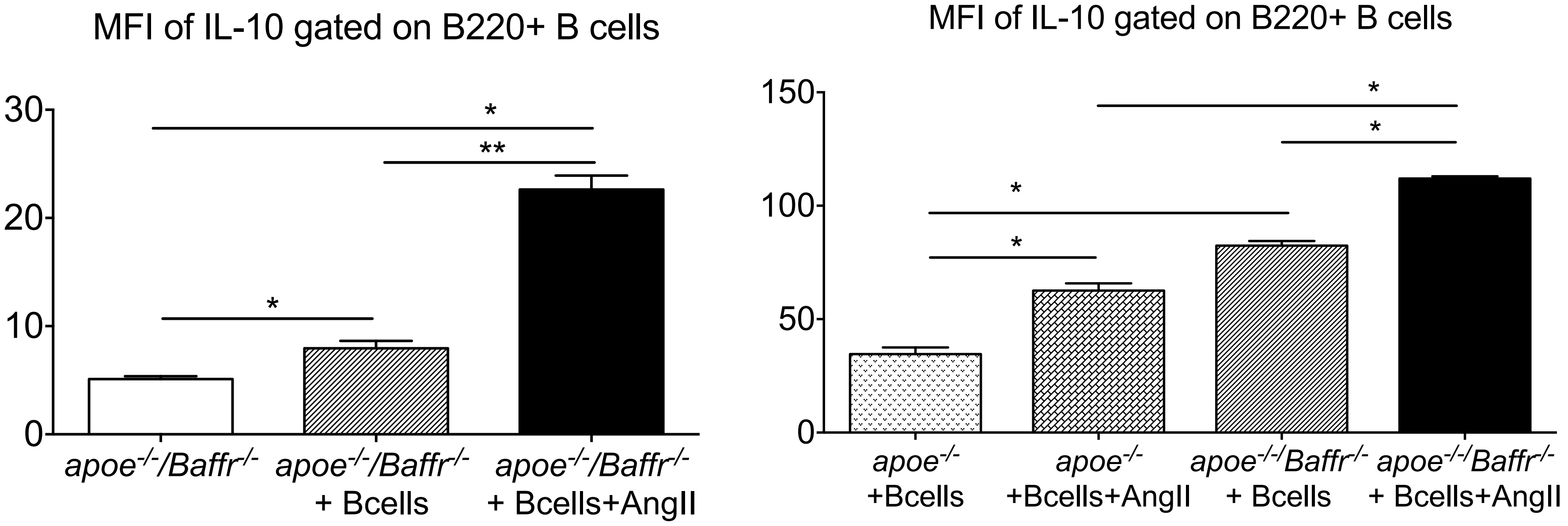

Facs staining on isolated B cells following ex vivo stimulation. Quantitative analysis of flow cytometry staining of MFI of IL-10 within B cells gated on B220+ cells in *apoe*$^{-/-}$/*Baffr*$^{-/-}$ mice with or without adoptive B cell transfer, infused with AngII for 7 days and their age matched controls without AngII infusion, n=6/group (a). Quantitative analysis of facs staining of MFI of IL-10 within B cells gated on B220+ cells in *apoe*$^{-/-}$ *or apoe*$^{-/-}$/*Baffr*$^{-/-}$ mice with adoptive B cell transfer, infused with AngII for 7 days and their age matched controls without AngII infusion, n=6/group (b). *P<0.05, **P<0.01

Supplementary Fig. 13

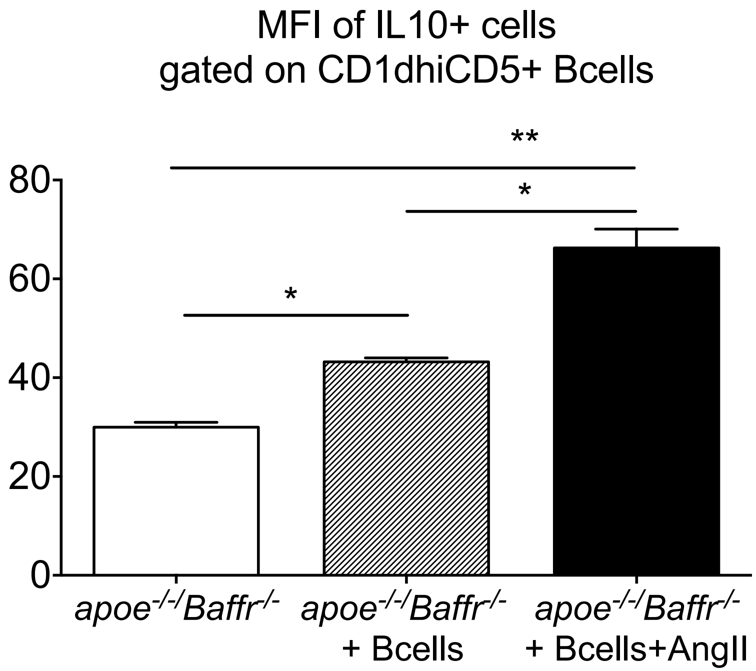

Quantitative analysis by flow cytometry staining of IL-10 production (MFI) by Bregs cells (defined as B220+CD1dhighCD5+ cells) in *apoe*$^{-/-}$/*Baffr*$^{-/-}$ mice with or without adoptive B cell transfer, infused with PBS or AngII for 7 days. n=6/group. *P<0.05, **P<0.01

Supplementary Fig. 14

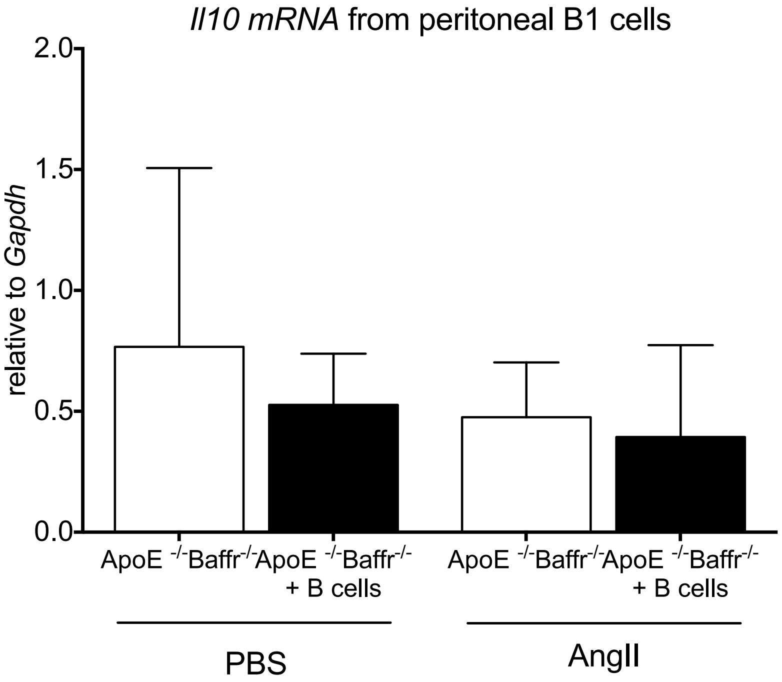

Ten-week old animals were infused with PBS or AngII (1µg/kg/min) during 28 days. B cell resplenishment (30.10^6 cells/mouse) was done 3 weeks before PBS/AngII infusion. At sacrifice $CD19^+CD11b^+$ B1 peritoneal cells were sorted and *IL-10 mRNA* expression was analyzed by qPCR. There was no difference of *IL-10 mRNA* expression between groups. N=4-5/group.

Supplementary Fig. 15

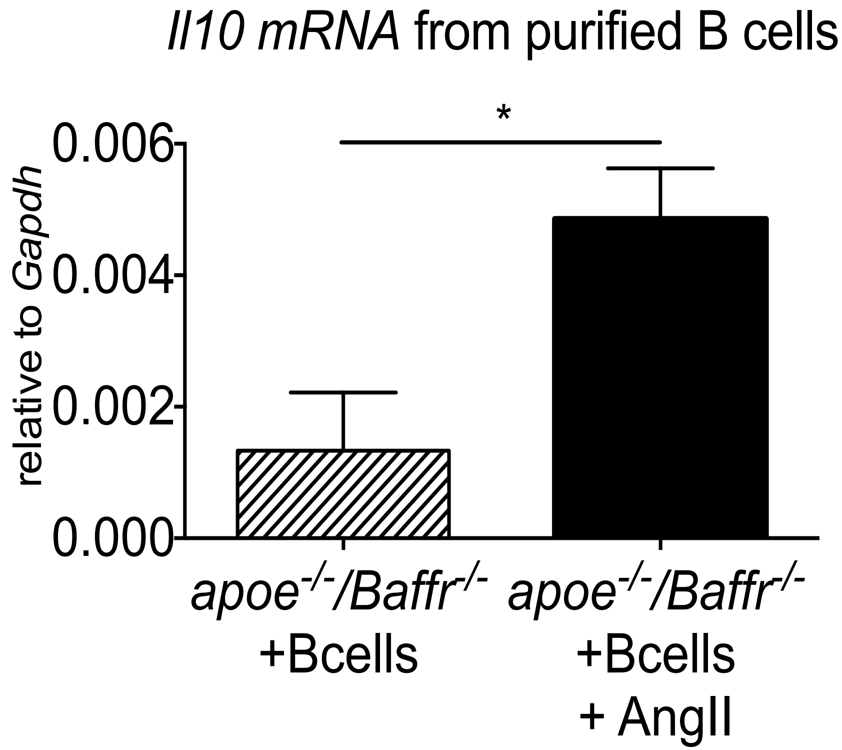

Quantitative analysis by qPCR of IL-10 mRNA expression by purified splenic B cells from *apoe*$^{-/-}$/*Baffr*$^{-/-}$ mice resupplemented with B cells, infused with PBS or AngII for 7 days. N=4-5/group. *P<0.05

Supplementary Fig. 16

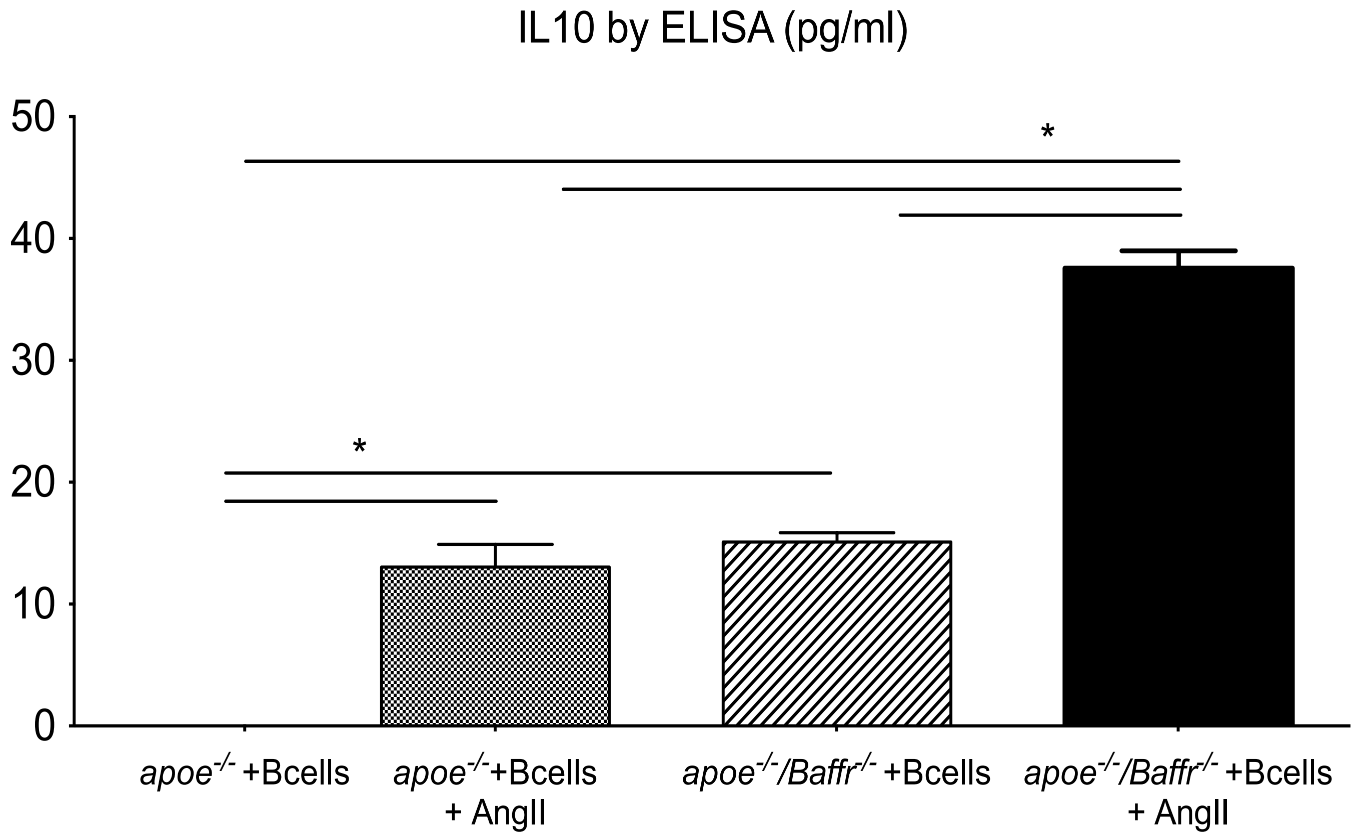

Splenic B cells isolated from *apoe*$^{-/-}$ and *apoe*$^{-/-}$/*Baffr*$^{-/-}$ mice adoptively transferred with B cells with or without AngII infusion for 7 days were stimulated *ex vivo* with α-CD40/α-IgM. IL10 levels in the supernatant were determined by ELISA. n=6/group. *$p<0.05$.

Supplementary Fig. 17

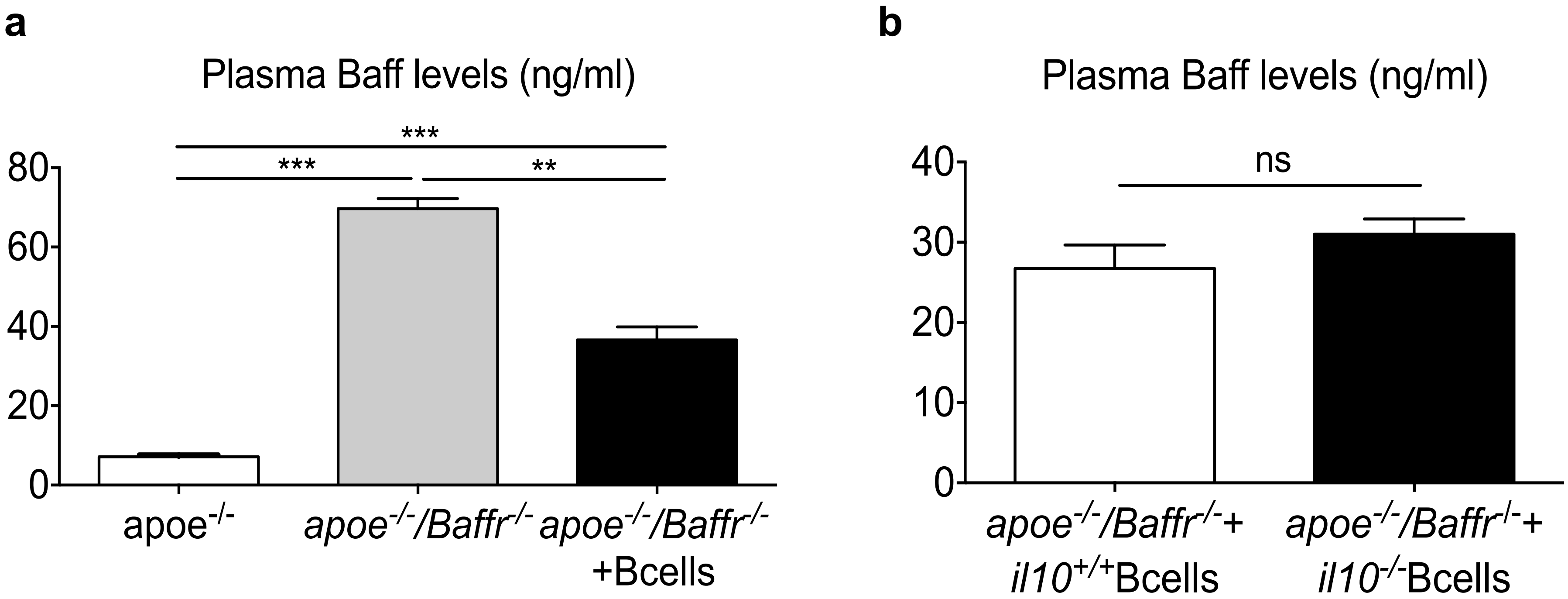

ELISA measurements of soluble BAFF levels in the plasma. (a) Plasma levels of soluble BAFF shows significant increase in *apoe*$^{-/-}$/*Baffr*$^{-/-}$ mice compared to *apoe*$^{-/-}$ *and apoe*$^{-/-}$/*Baffr*$^{-/-}$ mice replenished with B cells, n=8/group. B, The plasma BAFF levels were not different between *apoe*$^{-/-}$/*Baffr*$^{-/-}$ mice replenished with Il-10$^{+/+}$B cells compared to *apoe*$^{-/-}$/*Baffr*$^{-/-}$ mice replenished with IL-10$^{-/-}$ B cells, n=6-8/ group. **p<0.01,***p<0.001 and ns denotes non significance.

Supplementary Fig. 18

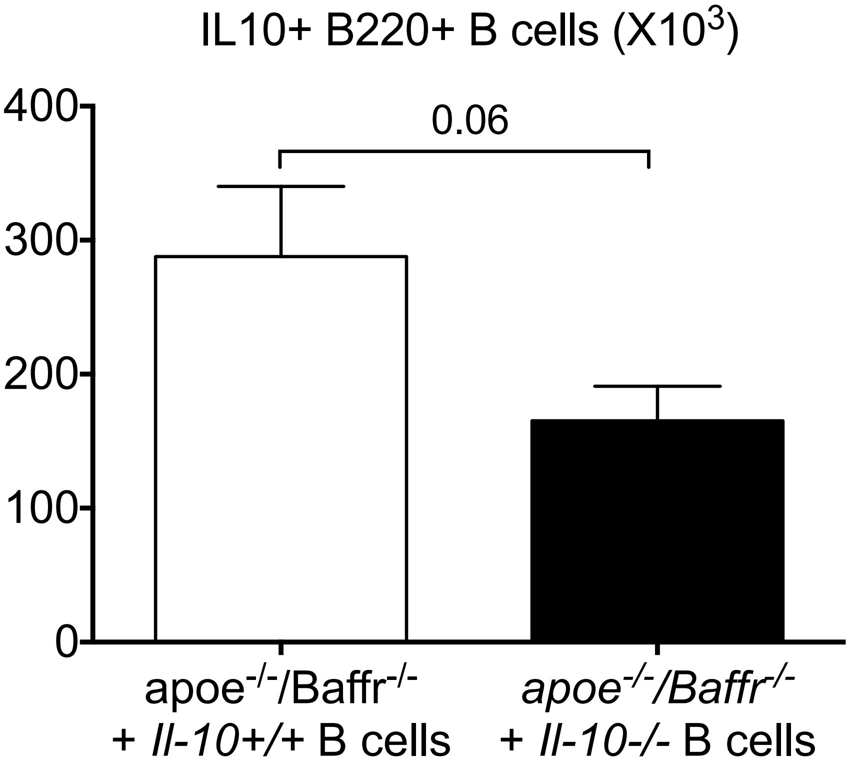

Quantification of splenic IL-10-producing B cell absolute number in *Apoe*$^{-/-}$*/Baffr*$^{-/-}$ mice supplemented with *Il-10+/+ or Il-10-/-* B cells and infused with AngII during 28 days. N=6-8 mice/group

Supplementary Fig. 19

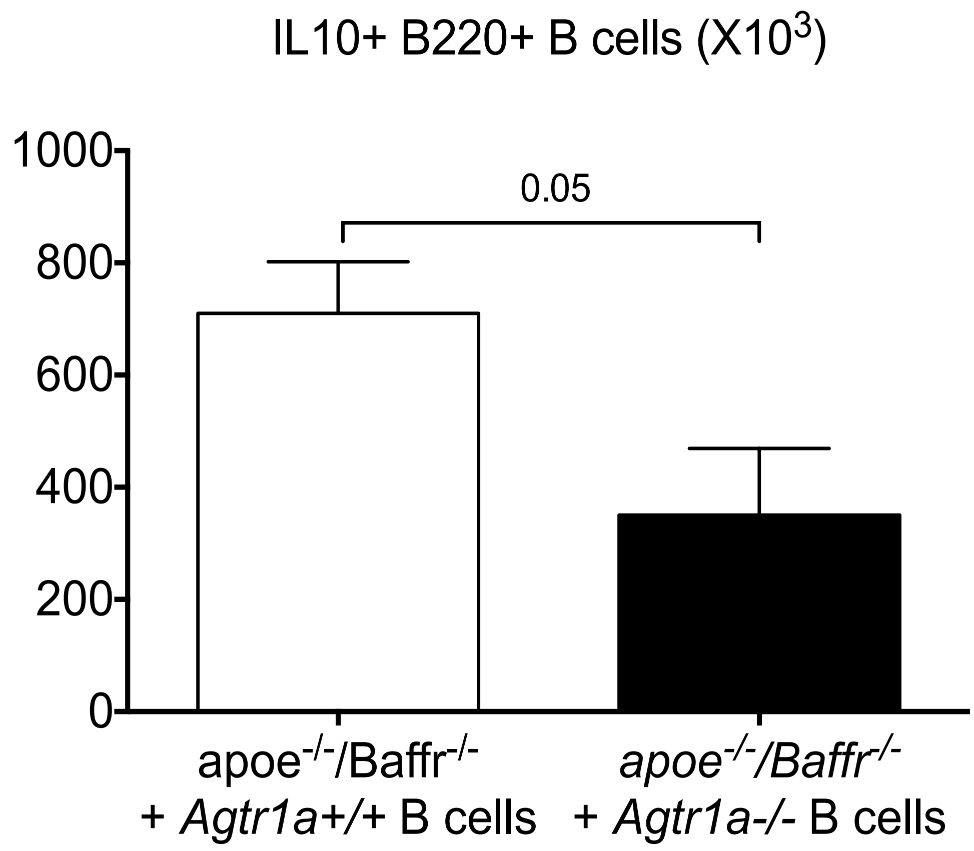

Quantification of splenic IL-10-producing B cell absolute number in *Apoe*$^{-/-}$*/Baffr*$^{-/-}$ mice supplemented with *Agtr1a+/+ or Agtr1a-/-* B cells and infused with AngII during 28 days. N=8 mice/group

Supplementary Fig. 20

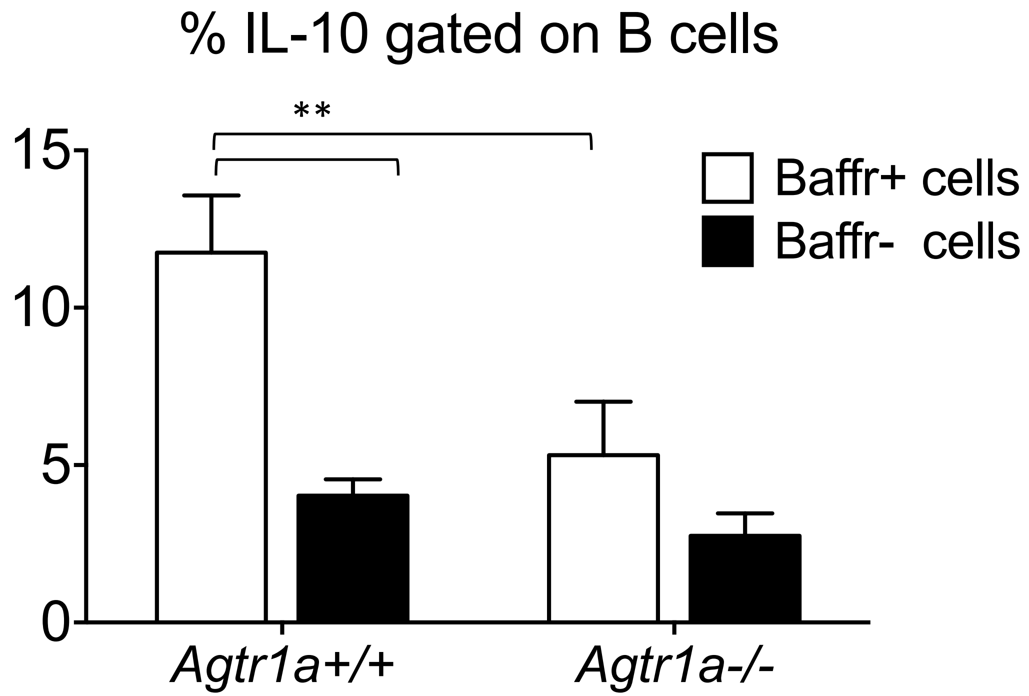

Splenic B cells isolated from *Agtr1a*$^{+/+}$ *or Agtr1a*$^{-/-}$ were adoptively transferred in *apoe*$^{-/-}$/*Baffr*$^{-/-}$ mice that were infused with AngII during 28 days. Resident immature B cells are defined as BAFFr- and transferred mature B cells as Baffr+. As shown in the figure, the transferred Baffr+ B cells produce much more IL-10 than resident immature Baffr- B cells, through Agtr1a activation. N=8/group. **$p<0.01$.
